# Mechanistic Study into Free Radical-Activated Glycan Dissociations through Isotope-Labeled Cellobioses

**DOI:** 10.1021/acs.analchem.2c04649

**Published:** 2023-01-30

**Authors:** Kimberly Fabijanczuk, Zaikuan Josh Yu, Rose M. Bakestani, Rayan Murtada, Nicholas Denton, Kaylee Gaspar, Tara Otegui, Jose Acosta, Hilkka I. Kenttämaa, Henk Eshuis, Jinshan Gao

**Affiliations:** † Department of Chemistry and Biochemistry, Montclair State University, 1 Normal Avenue, Montclair, New Jersey 07043, United States; ‡ Department of Chemistry, Purdue University, 560 Oval Drive, West Lafayette, Indiana 47907, United States

## Abstract

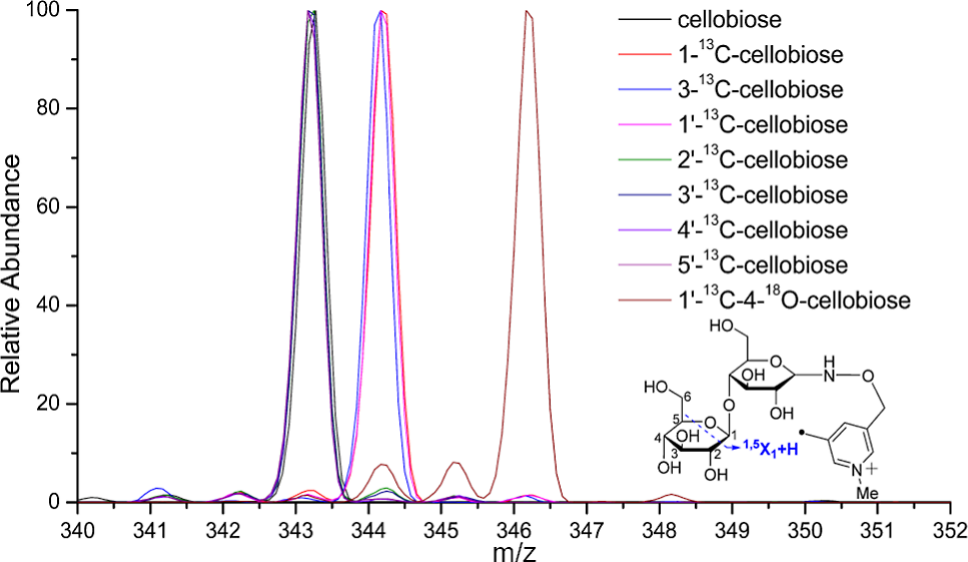

Inspired by the electron-activated dissociation technique, the most potent tool for glycan characterization, we recently developed free radical reagents for glycan structural elucidation. However, the underlying mechanisms of free radical-induced glycan dissociation remain unclear and, therefore, hinder the rational optimization of the free radical reagents and the interpretation of tandem mass spectra, especially the accurate assignment of the relatively low-abundant but information-rich ions. In this work, we selectively incorporate the ^13^C and/or ^18^O isotopes into cellobiose to study the mechanisms for free radical-induced dissociation of glycans. The eight isotope-labeled cellobioses include 1-^13^C, 3-^13^C, 1′-^13^C, 2′-^13^C, 3′-^13^C, 4′-^13^C, 5′-^13^C, and 1′-^13^C–4-^18^O-cellobioses. Upon one-step collisional activation, cross-ring (X ions), glycosidic bond (Y-, Z-, and B-related ions), and combinational (Y_1_ + ^0,4^X_0_ ion) cleavages are generated. These fragment ions can be unambiguously assigned and confirmed by the mass difference of isotope labeling. Importantly, the relatively low-abundant but information-rich ions, such as ^1,5^X_0_ + H, ^1,4^X_0_ + H, ^2,4^X_0_ + H–OH, Y_1_ + ^0,4^X_0_, ^2,5^X_1_-H, ^3,5^X_0_-H, ^0,3^X_0_-H, ^1,4^X_0_-H, and B_2_–3H, are confidently assigned. The mechanisms for the formations of these ions are investigated and supported by quantum chemical calculations. These ions are generally initiated by hydrogen abstraction followed by sequential β-elimination and/or radical migration. Here, the mechanistic study for free radical-induced glycan dissociation allows us to interpret all of the free radical-induced fragment ions accurately and, therefore, enables the differentiation of stereochemical isomers. Moreover, it provides fundamental knowledge for the subsequent development of bioinformatics tools to interpret the complex free radical-induced glycan spectra.

## Introduction

The significance of glycans has been established in various aspects of health, such as cancer metastasis, Alzheimer’s disease, inherited diseases, pathogen-host interactions, and immune recognition.^1−5^ However, the structural complexity and diversity of glycans pose a major analytical challenge to glycan structural analysis. Recently, with the advent of electron-activated dissociation (ExD) techniques, mass spectrometry (MS) has become the most powerful tool for glycan structural elucidation.^6^ ExD is a general term describing an electron-involved and radical-driven dissociation technique, including electron capture dissociation (ECD),^7−10^ negative-ion ECD,^11^ electron-transfer dissociation (ETD),^12^ negative ETD,^13^ electron detachment dissociation (EDD),^14−16^ electron ionization dissociation (EID),^7,17^ and electronic excitation dissociation (EED).^18−21^ ECD and ETD require multiply charged precursors to generate a radical and at least one charge site. EDD applies to the analysis of multiple negatively charged glycans, such as glycosaminoglycans (GAGs) and sialylated glycans. EED and EID can be used to analyze singly and multiply charged glycans in the positive ion mode. Besides ExD, ultraviolet photodissociation (UVPD) and collision-induced dissociation (CID) of highly labile radical precursors have been developed to generate free radicals, which instantaneously directs glycan dissociations in the gas phase.^22−25^

Tremendous efforts have been devoted to performing mechanistic studies of free radical-induced fragmentation of peptides and proteins.^26−36^ In contrast, very little attention has been paid to the mechanisms of free radical-induced glycan dissociations. It has been previously reported that glycan dissociations involve the generation of a nascent free radical followed by a hydrogen or hydroxyl abstraction and complex migration and rearrangement.^9,10,20,37,38^ Lin et al. investigated the ECD fragmentation mechanisms for the Mg^2+^-adducted glycans by using cellobiose-Mg^2+^ as the model system.^10^ A low-energy (∼1.5 eV) electron was discovered to being captured by Mg^2+^ to form Mg^•+^, which abstracts a hydroxyl group from the glycan moiety to generate a carbon radical followed by subsequent free radical migration and free radical-induced α-cleavage to produce various glycosidic and cross-ring fragment ions. Huang et al. used cellobiose-Na^+^ as the model system to investigate the EED mechanisms for metal-adducted glycans under irradiation of electrons with energy exceeding their ionization potential.^20^ First, the EED of cellobiose-Na^+^ was found to produce a mixture of radical cations and ring-opened distonic ions. Second, the distonic ions capture a low-energy electron to produce diradicals with trivial singlet-triplet splitting. Finally, the triplet diradicals undergo sequential radical-induced α-cleavage to form a variety of fragment ions. They also found that the abundances of fragment ions depend on the stability of the distonic ions from which they originate. Amster et al. proposed the mechanism of EDD on GAGs, wherein a nascent free radical was generated by the detachment of an electron from the multiply charged precursor, followed by hydrogen abstraction from either a hydroxyl group or H–C_*x*_ to generate an oxygen radical or carbon radical, and finally the consecutive free radical-driven α-cleavages to produce various fragment ions.^14^

Inspired by ExD and UVPD, we recently developed an alternative method to generate free radical on singly charged glycans at the well-defined sites by CID on methylated free radical-activated glycan sequencing (Me-FRAGS) reagent-derivatized glycans.^24^ By locating the generation of free radical at the unique reducing terminus of glycans and the charge on the pyridine moiety of the reagent, the fragmentation efficiency was significantly increased and systematic and predictable fragment ions, including glycosidic bonds and cross-ring cleavages, were generated.^25,38^ The free radical-induced glycan dissociation by using Me-FRAGS is proposed to be initiated by hydrogen abstraction followed by sequential rearrangements. However, a lack of detailed understanding of the free radical-induced glycan fragmentation has hindered the rational optimization of the free radical reagents and the interpretation of the MS spectra, especially the accurate assignment of relatively low-abundant ions. Therefore, it is crucial to perform mechanistic studies on free radical-induced glycan fragmentations for the better design of Me-FRAGS reagents and the development of bioinformatics tools for complex free radical-induced glycan dissociation spectra. Here, to probe the mechanisms for free radical-induced glycan dissociations, we synthesized eight ^13^C and/or ^18^O isotope-labeled cellobioses. These eight cellobioses differ only in the locations of the ^13^C and/or ^18^O labeling on the cellobiose. This will, for the first time, allow detailed mechanistic studies for free radical-induced glycan dissociations. In addition, computational studies using density functional theory (DFT) and molecular mechanics simulations were performed to obtain a more in-depth understanding of the dissociation process, with particular emphasis on the initial hydrogen abstraction.

## Experimental Section

### Chemicals and Reagents

**Scheme 1:**
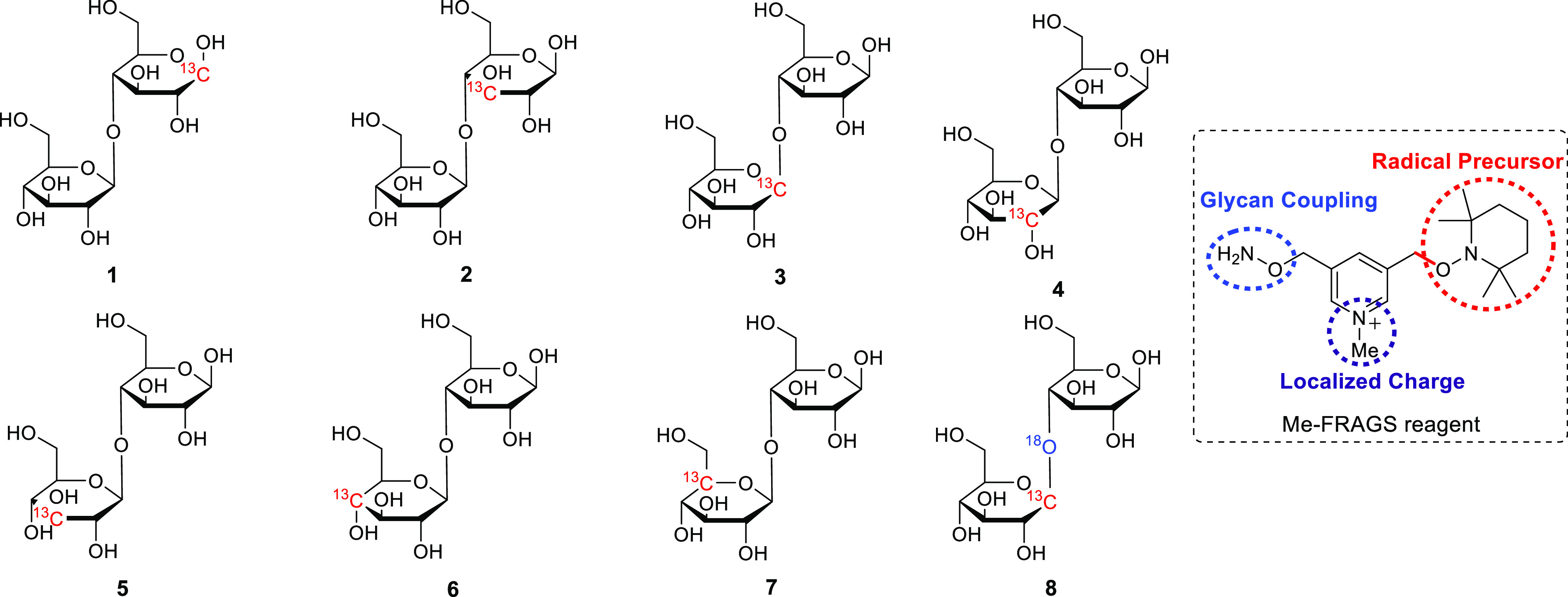
Structures of the ^13^C- and/or ^18^O-Labeled Cellobioses, and Me-FRAGS Reagent

[1-^13^C]-cellobiose and [1′-^13^C]-cellobiose (**1** and **3**, Scheme 1) were purchased from Omicron Biochemicals, Inc. (South Bend, IN, USA). The [1-^13^C]-cellobiose (**2**, Scheme 1), the ^13^C- and ^18^O-doubly-labeled cellobiose (Glc[1-^13^C]–^18^O-Glc-OH, **8**, Scheme 1), and 1-^13^C-labeled cellotriose were received from Dr. Kenttämaa’s group, and the synthesis and characterization of these two compounds have been published.^39,40^ All solvents are of HPLC grade and were purchased from EMD Merck (Gibbstown, NJ, USA). All other chemicals were purchased from Sigma-Aldrich (St. Louis, MO, USA). The synthesis of the Me-FRAGS reagent and glycan derivatization were achieved according to the previously reported procedures.^23,24^

### Synthesis of Isotope-Labeled Cellobiose

The synthesis protocol for ^13^C-labeled cellobioses (**4–7**, Scheme 1) is summarized in Scheme S1.^39−44^ Briefly, ^13^C-labeled glucose was acetylated with acetic anhydride to produce peracetylated glucose, the C_1_-acetylation was selectively deprotected to produce a free anomeric hydroxyl group at the C_1_ site, and the anomeric hydroxyl group was transformed into trichloroacetimidate to produce the end product of the ^13^C-labeled branch. For the unlabeled glucose unit, the anomeric acetyl group of the peracetlyated glucose was selectively converted to a benzyl group. The remaining acetyl groups were hydrolyzed to generate four free hydroxyl groups at the C_2_, C_3_, C_4_, and C_6_ sites, where the hydroxyl groups at the C_4_ and C_6_ sites were temporally protected by reacting with benzaldehyde dimethyl acetal, the hydroxyl groups at the C_2_ and C_3_ sites were re-protected by acetylation, and the hydroxyl group at the C_4_ site was deprotected to produce the end product of the unlabeled branch. The ^13^C-labeled subunit (S3 in Scheme S1) and the unlabeled subunit (S8 in Scheme S1) reacted to form the glycosidic bond. Finally, all of the acetylation and benzyl protection groups were deprotected to generate the target ^13^C-labeled cellobiose (**2–7**, Scheme 1).

### Mass Spectrometry

A Thermo Fisher Scientific linear quadrupole ion trap (LTQ-XL) mass spectrometer (Thermo, San Jose, CA, USA) equipped with an electrospray ionization (ESI) source was employed in this study. The derivatized glycan sample solutions were directly infused into the ESI source of a mass spectrometer via a syringe pump at a flow rate of 5-10 μL/min. Critical parameters of the mass spectrometer include a spray voltage of 5–6 kV, capillary voltage of 30–40 V, capillary temperature of 275 °C, sheath gas (N_2_) flow rate of 10 (arbitrary unit), and tube lens voltage of 50–200 V. Other ion optic parameters were optimized by the auto-tune function in the LTQ-XL tune program for optimal signal intensity. CID was performed by resonance excitation of the selected ions for 30 ms. The normalized CID energy was 10–35 (arbitrary unit).

## Computational Section

### Transition State Optimization

**Figure 1:**
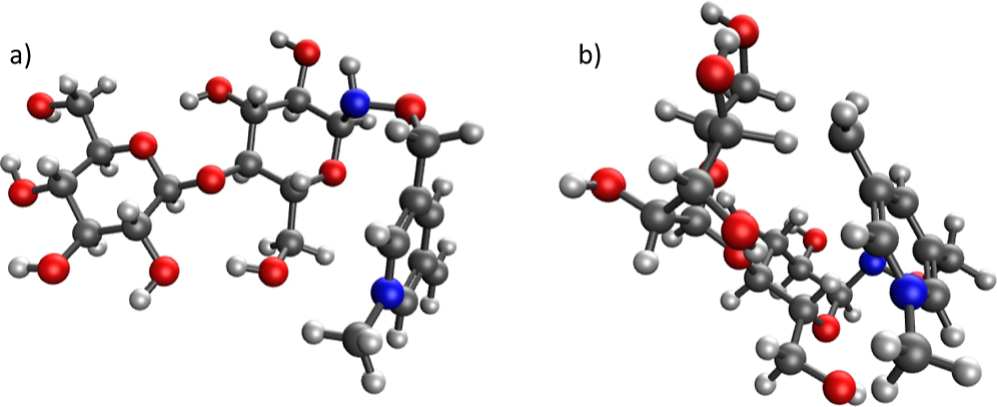
(a) Lowest energy conformer for the unconstrained Me-FRAGS cellobiose ions as obtained from CREST simulations. This structure was used as a reference to obtain barrier heights for the initial hydrogen abstraction step in Schemes 2, 3 and S2–S7. (b) Transition state structure for the hydrogen abstraction step in Scheme 2, which leads to the formation of the ^1,5^X_1_ + H ion.

The transition states for the intramolecular hydrogen-transfer reaction for the formation of the ions in Table 1 are found using a reaction path optimization method as implemented in the woelfling module^45^ in TURBOMOLE [tm7.6]. Starting with an initial and final guess structure, a reaction path was optimized by using the default settings. If the resulting path was not converged, the two minimum energy structures were used to recompute the path. The highest energy structure along the path was taken as the initial guess for the transition state search, which was performed at the DFT level. The optimized transition states were verified to have only one imaginary frequency corresponding to the reaction coordinate through the computation of the analytical hessian. To account for the structural flexibility of the molecule, the conformer-rotamer ensemble sampling tool (CREST) utility/driver^46,47^ for the xtb program^48^ was used to perform conformational sampling for all optimized transition states to identify conformers. The three atoms involved in the hydrogen transfer were kept frozen (i.e., the acceptor, donor, and hydrogen atom were fixed), whereas all other atoms were included in the metadynamics. The five conformers with the lowest energies for each reaction path were reoptimized at the DFT level using a transition state search algorithm and the resulting transition states were verified with a hessian calculation. For these optimized structures, single-point energy calculations were performed with larger basis sets and higher level functionals. The lowest energy structure was used to obtain barrier heights and relative energies of the transition states. See Figure 1 for representative structures. The details about the reaction barriers and computational methodology are described in the Supporting Information.

**Table 1:** Relative Electronic Energies and Barrier Heights (in kcal/mol) for the Transition States for the Sequential Hydrogen Abstraction for the Different Fragmentation Schemes Presented in Schemes 2, 3, and S2–S7^a^

scheme	ion	relative energy	barrier height
2	^1,5^X_1_ + H	0	18.2
S2	^2,5^X_1_-H	7.2	25.4
S3	^3,5^X_1_-H	9.5	27.7
S4	Y_1_ + 2H	4.6	22.8
S5	Y_1_	18.8	37.0
S6	Y_1_ + ^0,4^X_0_	10.3	28.5
S7	Z_1_	4.1	22.3
3	Z_1_ + H	15	33.2

a The energies were obtained with the PBE0 functional and Grimme’s D3 dispersion correction using the def2-TZVPP basis set. See the Computational Methods section, for full details on the calculations and how transition state structures were obtained.

## Results and Discussion

### CID of the Me-FRAGS Derivatized Cellobioses

**Figure 2:**
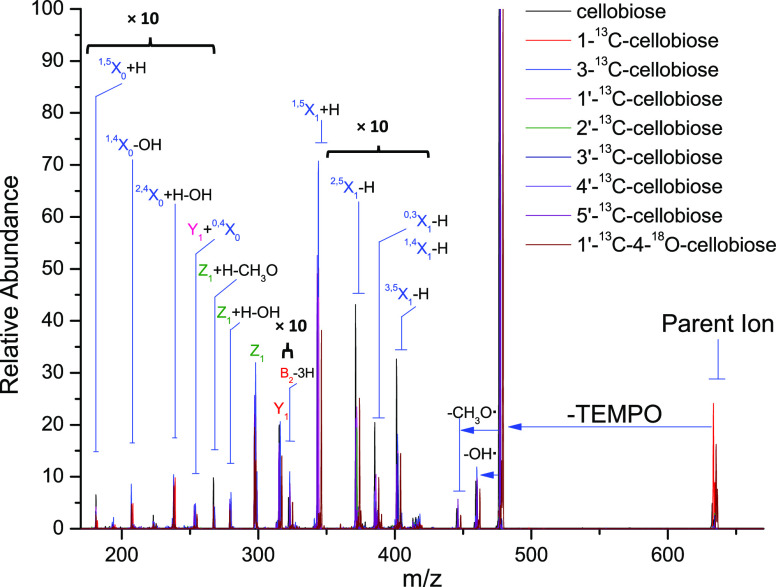
CID spectra of the eight Me-FRAGS-derivatized ^13^C/^18^O-labeled cellobioses and one Me-FRAGS-derivatized unlabeled cellobiose.

The combined CID spectra of Me-FRAGS-derivatized cellobioses are shown in Figure 2. The fragment ions are classified according to the Domon and Costello nomenclature.^49^ The high-abundance product ions, including ^1,5^X_1_ + H, Y_1_, Z_1_, Z_1_ + H–H_2_O, Z_1_ + H–CH_3_O, −TEMPO, −(TEMPO + OH^•^), and −(TEMPO + CH_3_O^•^), have been clearly assigned in previous studies.^23,24,50^ Besides these product ions, the product ions with relatively low abundances (×10 in Figure 2), including ^1,5^X_0_ + H, ^1,4^X_0_-OH, ^2,4^X_0_ + H–OH, Y_1_ + ^0,4^X_0_, B_2_–3H, ^2,5^X_0_-H, ^0,3^X_0_-H, and/or ^1,4^X_0_-H, and ^3,5^X_0_-H, for the first time are also successfully assigned and confirmed. Therefore, this provides much more structural information for glycan characterization and allows glycan isomer differentiation to be unambiguous and straightforward. The mechanisms, quantum chemical calculations, and verification of the assignment of all the product ions are described in the following sections.

### Cross-Ring Cleavage Ions: ^1,5^X_1_ + H, ^2,5^X_1_-H, ^3,5^X_1_-H, ^1,5^X_0_ + H, ^2,4^X_0_ + H–OH, ^1,4^X_0_-OH, ^0,3^X_0_-H, and ^1,4^X_0_-H Ions

**Figure 3:**
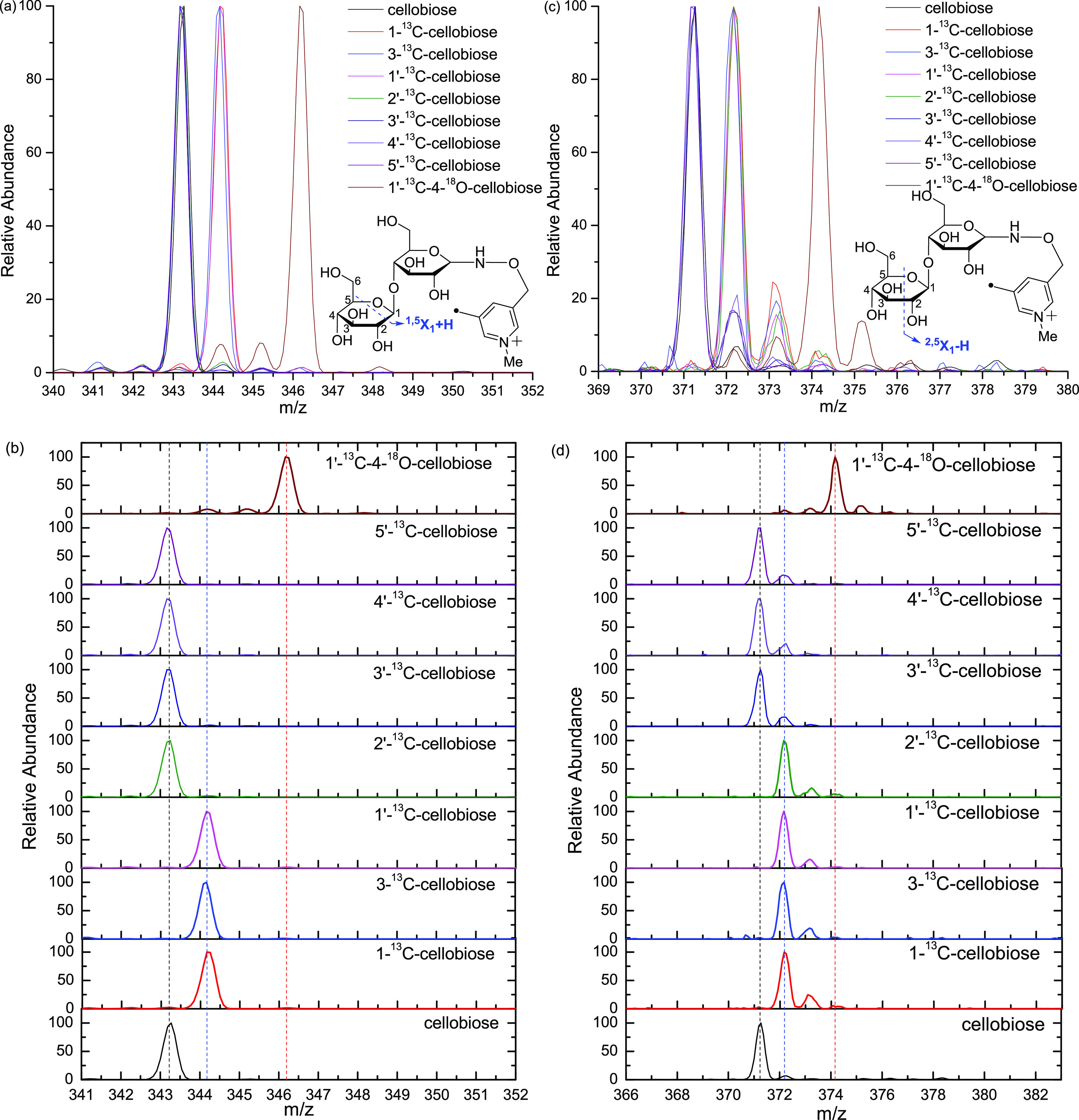
Zoom-in views (a, 340–352, ^1,5^X_1_ + H), stack views (b, 340–351, ^1,5^X_1_ + H), zoom-in views (c, 369–380, ^2,5^X_1_-H), and stack views (d, 368–380, ^2,5^X_1_-H) of CID spectra of the seven Me-FRAGS-derivatized ^13^C/^18^O-labeled cellobioses and Me-FRAGS-derivatized unlabeled cellobiose.

**Scheme 2:**
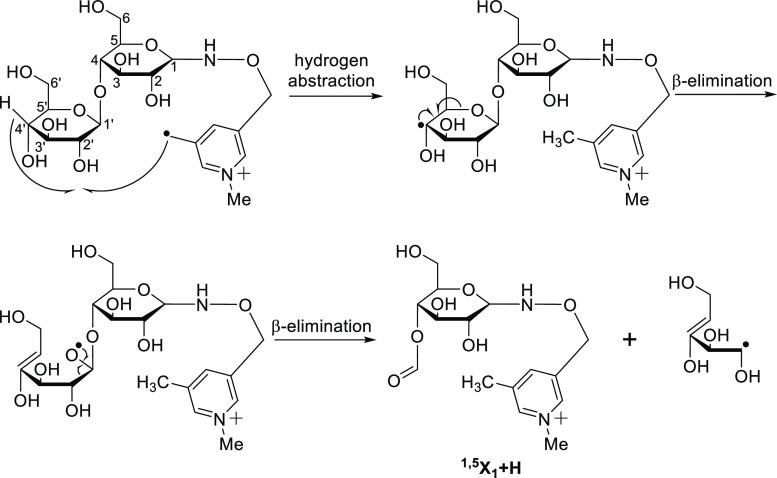
Mechanism for the Formation of ^1,5^X_1_ + H Ion

The systematic cross-ring fragments are crucial for linkage determination, differentiation of isomeric glycoforms, and determination of antennae substitutions for highly branched glycans. Only the most abundant ^1,5^X ion was confidently assigned in our previous report, while the other relatively low-abundant cross-ring cleavage ions were undiscussed due to the lack of information.^21,23,24^ Here, we assigned all these cross-ring cleavage ions, which are confirmed by the study of the ^13^C- and/or ^18^O-labeled cellobioses as detailed below. As shown in the zoom-in and stack views in Figure 3, the ^1,5^X_1_ + H ions of 2′-^13^C, 3′-^13^C-, 4′-^13^C-, and 5′-^13^C-cellobioses have the same mass as that of the unlabeled cellobiose because these four ^13^C-labeled cellobioses have the ^13^C isotope on the leaving side of this cross-ring cleavage. The ^1,5^X_1_ + H ions of 1′-^13^C-, 1-^13^C-, and 3-^13^C-cellobioses have a mass increase of 1, while the ^1,5^X_1_ + H ion of 1′-^13^C–4-^18^O-cellobiose has a mass increase of 3, compared to that of the unlabeled cellobiose. This is due to the fact that the ^13^C and/or ^18^O atoms of these four isotope-labeled cellobioses are on the remaining side of the cleavage. The assignment of ^1,5^X + H ion can also be verified by the formation of the ^1,5^X_0_ + H ion (Figure S1). Only the ^1,5^X_0_ + H ion of 1-^13^C-cellobiose has a mass increase of 1 as it has the ^13^C on the remaining side of the cleavage. The ^1,5^X + H ion is formed by hydrogen abstraction followed by β-elimination, as detailed in the subsequent discussion. In the first step of ^1,5^X ion formation, the nascent free radical generated by the loss of TEMPO abstracts a hydrogen atom from C_4_′ on the leaving side to generate a carbon-centered radical. In the second step, the resulting carbon-centered radical promotes β-elimination to form oxygen-centered radical and a double bond between C_4_′ and C_5_′ (Scheme 2). In the third step, the oxygen-centered radical induces further β-elimination to form the ^1,5^X + H ion.

As shown in Figure 3, the ^2,5^X_1_-H ions of cellobioses with ^13^C and/^18^O on the remaining residue, including 1-^13^C-, 3-^13^C-, 1′-^13^C-, 3′-^13^C-, and 1′-^13^C–4-^18^O-cellobioses, have a mass increase of 1 or 3 accordingly. Similarly, the ^3,5^X_1_-H ions of cellobioses with ^13^C and/or ^18^O on the remaining residue have the corresponding mass increase of 1 or 3 (Figure S1). The ^2,5^X_1_-H ion is formed by hydrogen abstraction from the hydroxyl group at the C_2_′ position, β-elimination to form a carbonyl group on the C_2_′ and a carbon-centered radical on the C_3_′, radical migration between C_3_′ and C_1_′ to form a carbon-centered radical on the C_1_′, and finally β-elimination to form another carbonyl group on C_1_ (Scheme S2). Similarly, ^3,5^X_1_-H ion (Figure S1) is generated by hydrogen abstraction from the hydroxyl group on C_3_′ followed by β-elimination, radical migration, and the second β-elimination (Scheme S3). The abundance of the ^2,5^X_1_-H and ^3,5^X_1_-H ions is much lower than that of the ^1,5^X_1_ + H ion. This can be rationalized by considering the transition state energy for the formation of these ions: the ^1,5^X_1_ + H ion has an 18.2 kcal/mol transition state energy, while ^2,5^X_1_-H and ^3,5^X_1_-H ions have 25.4 and 27.7 kcal/mol transition state energies, respectively (Table 1). Assuming Arrhenius kinetics, an increase of ∼7–10 kcal/mol in the barrier height reduces the rate constant for the process by at least an order of magnitude, which corresponds to a much lower abundance for the ^5^X_1_-H and ^3,5^X_1_-H ions. Similarly, the ^2,4^X_0_ + H–OH and ^1,4^X_0_-OH ions (Figure S2) are formed by sequential hydrogen abstraction, β-elimination, radical migration, and/or hydroxyl migration, as described in Schemes S8 and S9. It is quite common to see two different ions sharing the same *m*/*z* ion, such as the ^0,3^X_1_-H and ^1,4^X_1_-H ions. As shown in Figure S3, 1′-^13^C-, 1-^13^C-, and 3-^13^C-cellobioses have a mass increase of 1, while 1′-^13^C–4-^18^O-cellobiose has a mass increase of 3 for this cleavage, indicating that the cleavage is on the non-reducing terminus and C_1_′ is on the remaining residue of the cleavage. However, 2′-^13^C-, 3′-^13^C-, and 5′-^13^C-cellobioses have two peaks for this cleavage, wherein one has the same mass as the unlabeled cellobiose, while the other one has a mass increase of 1, indicating the presence of two types of cleavages, ^0,3^X_1_-H and ^1,4^X_1_-H.

### Y_1_, Y_1_ + 2H, and Y_1_ + ^0,4^X_0_ Ions

Systematic and predictable Y ions are critical for the determination of glycan topology. Here, Y (Y_1_ and Y_1_ + 2H) and Y + ^0,4^X_0_ ions are generated upon collisional activation of the Me-FRAGS-derivatized cellobioses. The assignments of these three ions are confirmed, and their formation mechanisms are described in detail below.

**Figure 4:**
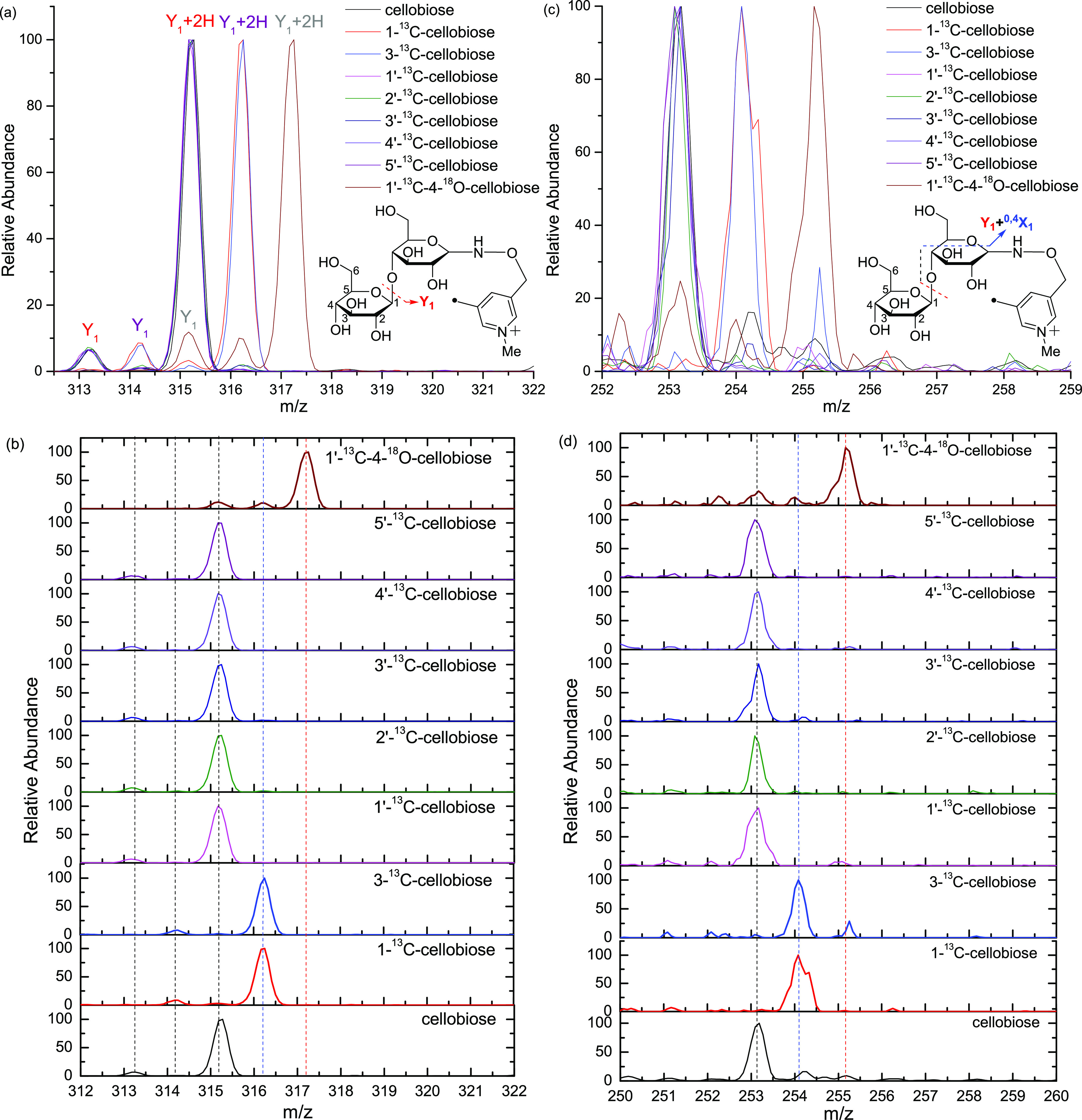
Zoom-in views (a, 312–322, Y_1_), stack views (b, 312–322, Y_1_), zoom-in views (c, 252–259, Y_1_ + ^0,4^X_0_), and stack views (d, 250–259, Y_1_ + ^0,4^X_0_) of CID spectra of the seven Me-FRAGS derivatized ^13^C/^18^O labeled cellobioses and Me-FRAGS derivatized unlabeled cellobiose.

Two types of Y ions (Y_1_ and Y_1_ + 2H) are generated via a free radical-initiated mechanism (a and b in Figure 4), although the relative abundance of Y_1_ + 2H is much higher than Y_1_.^24^ The assignments of the Y_1_ and Y_1_ + 2H ions are confirmed by a mass increase of 1 for the cellobiose with the ^13^C label on the reducing terminal subunit (1-^13^C- and 3-^13^C-cellobioses) and a mass increase of 2 for the 1′-^13^C–4-^18^O-cellobiose, which has the ^18^O in the middle (glycosidic bond) and ^13^C on the non-reducing terminal subunit. All the other isotope-labeled cellobioses share the same Y ions as the unlabeled cellobiose, thereby indicating the cleavage of the C_1_′–O glycosidic bond. The Y_1_ + 2H ion is generated via hydrogen abstraction from the C_2_′ on the leaving side followed by β-cleavage to form a reaction intermediate comprised the leaving residue with a double bond and a remaining residue with a highly reactive oxygen-centered radical, and finally the oxygen-centered radical on the remaining residue abstracts the second hydrogen from the leaving residue (Scheme S4). The Y_1_ ion is formed by hydrogen abstraction from the C_4_ site on the remaining side, followed by β-elimination to form a carbonyl group at the C_4_ site (Scheme S5). The abundance of the Y_1_ + 2H ion is much higher than that of the Y_1_ ion, which agrees with a significantly lower computed transition state energy for the formation of the Y_1_ + 2H ion than that of the Y_1_ ion, namely, 22.8 versus 38.0, respectively (Table 1).

Although the intensity of the Y_1_ + ^0,4^X_0_ ion is relatively low, it provides valuable structural information for the characterization of glycans. As shown in Figure 4, the Y_1_ + ^0,4^X_0_ ion of 1′-^13^C–4-^18^O-cellobiose has a mass increase of 2, clearly indicating the cleavage of the C_1_′-O glycosidic bond. The mass increase of the Y_1_ + ^0,4^X_0_ of 1-^13^C- and 3-^13^C-cellobioses indicates that this product ion contains C_1_ and C_3_ on the reducing terminus (Figure 4). Therefore, the Y_1_ + ^0,4^X_0_ ion is generated by a hydrogen abstraction from C_2_ of the reducing terminal glycan subunit, β-elimination to form a double bond between C_1_ and C_2_ and oxygen-centered radical, β-elimination to form a carbonyl group and a radical at the C_4_, followed by β-elimination to break the C_1_′–O glycosidic bond and form a carbonyl group at C_4_ (Scheme S6).

### Z_1_, Z_1_ + H, Z_1_ + H–OH, and Z_1_ + H–CH_3_O Ions

**Figure 5:**
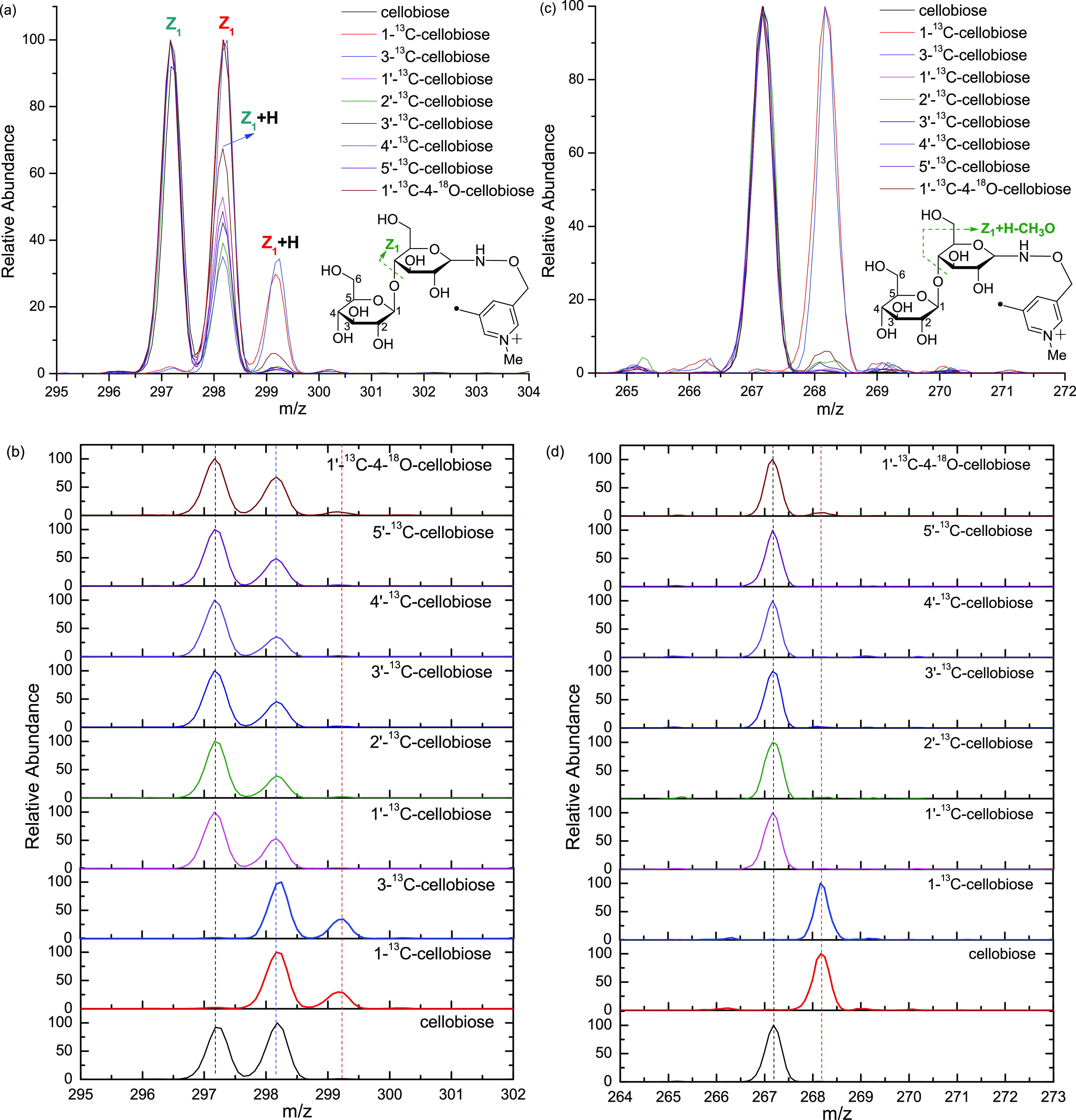
Zoom-in views (a, 295–304, Z_1_), stack views (b, 295–302, Z_1_), zoom-in views (c, 265–272, Z_1_ + H–CH_3_O), and stack views (d, 264–273, Z_1_ + H–CH_3_O) of CID spectra of the seven Me-FRAGS-derivatized ^13^C/^18^O labeled cellobioses and Me-FRAGS-derivatized unlabeled cellobiose.

**Scheme 3:**
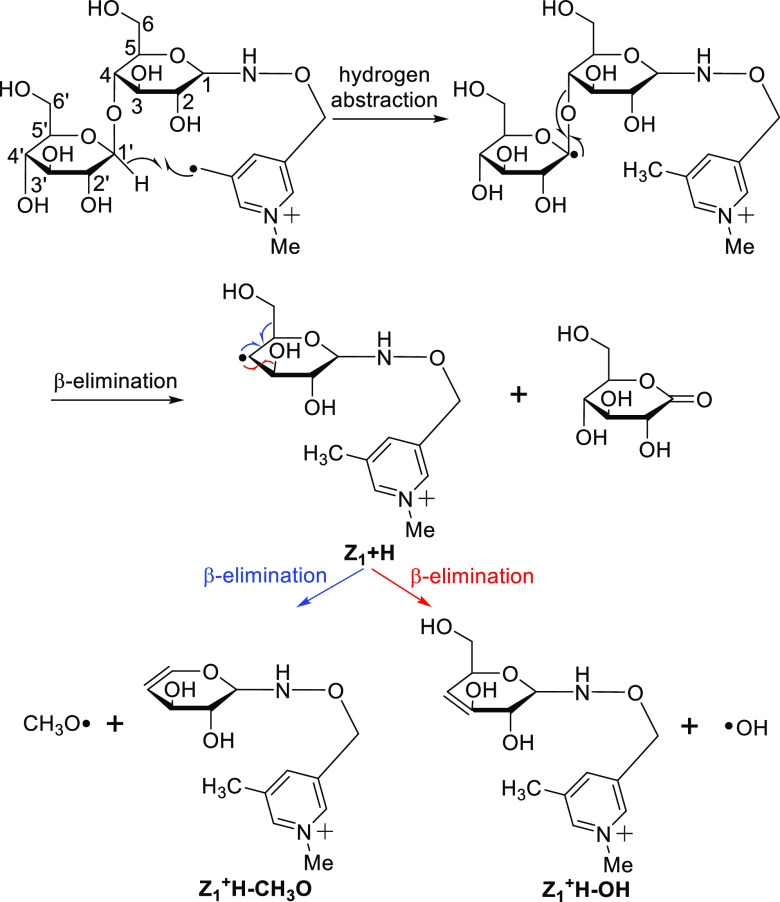
Mechanism for the Formation of Z_1_ + H, Z_1_ + H–OH, and Z_1_ + H–CH_3_O Ions

Similar to Y ions, the Z ions are generated via the cleavage of the O–C_1_ glycosidic bond, and therefore also provide valuable complementary information for glycan topology. Two types of Z ions (Z_1_ and Z_1_ + H) are generated via free radical-initiated mechanisms (a and b in Figure 5). Only the cellobioses with isotope labeling on the reducing terminal glycan subunit, 1-^13^C- and 3-^13^C-cellobioses, generate Z_1_ and Z_1_ + H ions with a mass increase of 1, while all the other isotope-labeled cellobioses share the same Z ions as the unlabeled cellobiose. The Z_1_ ion is generated via hydrogen abstraction from the C_3_ on the remaining side followed by β-elimination to form a double between C_3_ and C_4_ (Scheme S7). Similarly, the Z_1_ + H ion is formed by hydrogen abstraction from the C_1_′ position on the leaving residue followed by β-elimination to form a carbonyl group on the leaving residue and the carbon-centered radical on C_4_ of the remaining residue (Scheme 3). The Z_1_ + H ion is a distonic radical ion with a fixed charge on the nitrogen atom of the pyridine moiety and a highly reactive carbon-centered radical on C_4_ of the remaining residue, which further dissociates into Z_1_ + H–OH and Z_1_ + H–CH_3_O. As shown in Scheme 3, Z_1_ + H–OH and Z_1_ + H–CH_3_O ions are formed by further β-cleavage to generate the OH^•^ and CH_3_O^•^ losses, respectively (Figures 5 and S3). This is also confirmed by the generation of Z_1_ + H–OH and Z_1_ + H–CH_3_O ions upon further collisional activation of the Z_1_ + H ion (Figure S4). Similarly, Z-OH and Z-CH_3_O ions have been reported to be generated upon electron excitation dissociation (EED) and are used as the characteristic ions to differentiate glycan isomers.^21^ Similar to the X and Y ions, a difference in relative abundance is observed for the Z_1_ and Z_1_ + H ions, with Z_1_ being the more abundant. Once again, this is in line with the computed transition state barriers, which are 22.3 kcal/mol for Z_1_ and 33.2 kcal/mol for Z_1_ + H.

### B_2_–3H Ions

The B_2_–3H ion of all the isotope-labeled cellobioses has the same mass shift as the pure isotope-labeled ones, indicating the cleavage of the C_1_–N bond (Figure S5). B_2_–3H ion is generated by hydrogen abstraction to form a radical at C_2_, then β-elimination to form a double bond between C_1_ and C_2_ and the nitrogen-centered radical, followed by hydrogen abstraction and hydride abstraction (Scheme S10).

### 1-^13^C-cellotriose

While the focus of this work was using cellobiose, a 1-^13^C-labeled cellotriose was synthesized to probe if the fragmentation observed is similar to that of cellobiose. Generally, similar fragment identities such as ^2,5^X and ^3,5^X cleavages as well as Z and Y cleavages along the numerous glycosidic bonds were observed (Figure S6). DFT calculations were not performed for this; however, we hypothesize that the mechanisms of fragmentation are reminiscent of those proposed for cellobiose. We further hypothesize that the fragmentation patterns observed for cellobiose and cellotriose can be more widely applicable to various glycans, especially larger glycans though would require further extensive studies for verification.

## Conclusions

The mechanisms of free radical-induced glycan dissociations were investigated by employing the ^13^C- and/or ^18^O-labeled cellobioses as a model system. It was found that a variety of fragment ions were generated upon one-step collisional activation via cascade radical-driven reactions, including hydrogen abstraction, β-elimination, radical migration, and hydride abstraction. The relatively high-abundance ions (^1,5^X_1_ + H, Y_1_, Z_1_, Z_1_ + H–OH, and Z_1_ + H–CH_3_O) are generally produced by hydrogen abstraction followed by sequential β-elimination. The formation of the relatively low-abundance ions (^2,5^X_1_-H, ^3,5^X_1_-H, ^2,4^X_0_ + H–OH, ^1,4^X_0_-OH, ^0,3^X_0_-H, and ^1,4^X_0_-H ions) are generally initiated by hydrogen abstraction followed by radical migration and β-elimination. Meanwhile, the mechanistic investigation revealed some unexpected fragment ions, such as Y_1_ + ^0,4^X_0_ and B_2_–3H, which provides extra valuable structural information. It needs to be noted, however, that the formation of Y_1_ + 2H and B_2_–3H ions involves a second hydrogen abstraction from the leaving residue. The trend in the relative abundance of the observed ions is in good agreement with the computed energy barriers for the initial hydrogen abstraction. This suggests that this initial step is rate-limiting and, therefore, controls the kinetics of the entire fragmentation process. Further development of free radical tags for simultaneous glycan characterization and quantitation is under investigation for the future application of this technique to complex biological samples.

## Supporting Information Available

Computational methodology, overview of synthesis for ^13^C-labeled cellobiose, fragmentation mechanisms, additional zoomed-in MS fragmentation spectra for ^1,5^X_0_ + H, ^3,5^X_1_-H, ^1,4^X_0_-OH, ^2,4^X_0_ + H–OH, CID spectrum of Me-FRAGS-derivatized 1-^13^C-cellotriose, synthesis procedures for all isotope-labeled cellobioses and cellotriose, relative electronic energies for transition states for sequential hydrogen abstractions for fragmentation mechanisms, and ^1^H NMR spectra of synthesized isotope-labeled cellobioses and cellotriose (PDF)
