# Supplementary material for: Mechanistic Study into Free Radical-Activated Glycan Dissociations through Isotope-Labeled Cellobioses
Source: Anal Chem. 2023 Jan 30;95(5):2932–41. doi: 10.1021/acs.analchem.2c04649 (PMC10129047; doi:10.1021/acs.analchem.2c04649)
Supplement: Supplementary file 1 — ac2c04649_si_001.pdf [file ac2c04649_si_001.pdf]

Supporting Information

Mechanistic Study into Free Radical Activated Glycan Dissociations through Isotope-Labeled Cellobioses

Kimberly Fabijanczuk,† Zaikuan Josh Yu,‡ Rose M. Bakestani,† Rayan Murtada,† Nicholas Denton,† Kaylee Gaspar,† Tara Otegui,† Jose Acosta,† Hilkka I. Kenttämaa,‡ Henk Eshuis,†* Jinshan Gao†*

† Department of Chemistry and Biochemistry, Montclair State University, 1 Normal Avenue, Montclair, NJ 07043, USA
‡ Department of Chemistry, Purdue University, 560 Oval Drive, West Lafayette, Indiana 47907, United States

Table of Contents

- Computational Methodology – **S2-S3**
- **Scheme S1.** Overview of synthesis for ^{13}C-labeled cellobiose – **S3**
- **Schemes S2-10.** Fragmentation Mechanisms- **S4-7**
- **Figures S1-5.** Additional zoomed-in MS fragmentation spectra for $^{1,5}X_0$+H, $^{3,5}X_1$-H, $^{1,4}X_0$-OH, $^{2,4}X_0$+H-OH – **S7-12**
- **Figures S6.** CID spectrum of Me-FRAGS derivatized 1-^{13}C-cellotriose – **S13**
- Synthesis procedures for all isotope-labeled cellobioses and cellotriose – **S14-18**
- **Table 1.** Relative electronic energies for transition states for sequential hydrogen abstractions for fragmentation mechanisms – **S19**
- References – **S20**
- ^{1}H NMR spectra of synthesized isotope-labeled cellobioses and cellotriose – **S212-36**

Computational Methodology

The reaction barrier for the hydrogen transfer was computed with respect to the overall minimum. Each reaction path is therefore assumed to start from the same structure. The rationale for this choice is that the molecule has a large amount of rotational flexibility and many conformers within a small energy range. The CREST conformer search resulted in over 500 unique conformers within a 6 kcal/mole energy window for the unconstrained structure and the transition state structures. Under the experimental conditions, the molecule is likely to possess some excess energy after the collision induced dissociation and is, therefore, able to move quite freely from one local minimum to another. It is unlikely that the molecule will be trapped in a local minimum for a long period of time. This is in agreement with what we observe for structure optimizations for the initial and final states of the Reaction Path Optimization calculations. Once the constraints of the reaction path are removed the initial structures relax and wander off on a very flat potential energy surface. The resulting optimized structure is often quite unlike the original initial structure. We feel therefore that it is justified to use a single overall minimum energy structure as a point of reference for all reaction paths. We further assume that the reaction kinetics are governed by the energy required to overcome the barrier and that all studied reaction paths are equally accessible to the radical CH_2 group. We have not seen evidence of steric hindrance for any of the reaction paths and it seems unlikely that large differences in sampling probability of conformational space exist. For example, the reactions proposed in **Schemes 2** and **S3** involve hydrogen transfer from atoms that are spatially quite close to each other and therefore most likely equally accessible. However, **Scheme 2** leads to fragments that are much more dominant in the MS spectrum than **Scheme S3** which suggests that barrier heights dominate the rate of the reaction.

For CREST, the iMTD-GC algorithm was used with the GFN2-xTB method with the default settings for the metadynamics.[1] The DFT and RPA calculations were performed using the ridft and rirpa modules from the TURBOMOLE program package. All DFT level structure optimizations were performed using the Perdew-Burke-Ernzerhof (PBE)[2] functional and the def2-SVP basis set.[3] The use of a generalized gradient approximation (GGA) functional and a small basis set allows for efficient optimizations and is known to yield reasonable structures.

Single-point calculations were performed with the def2-TZVPP basis set as it yields energies that are well converged for most functionals. The hybrid GGA PBE0 functional[4] and the hybrid meta-GGA PW6B95 functional,[5] both with Grimme's dispersion correction D3-BJ[6-7] were chosen.

These functionals are widely used and known to provide accurate energies. The Grimme correction incorporates contributions due to dispersion interactions. As an independent check, the parameter-free random phase approximation (RPA) method[8-10] was used (with the def2-QZVPP basis set[3] due to its slower basis set convergence). RPA naturally includes dispersion interactions and provides a balanced description of electronic interactions for main-group elements. Self-consistent PBE orbitals were used as input for the RPA calculation. Resolution-of-the-identity methods were employed to approximate the 4-center integrals in both DFT and RPA with auxiliary basis sets[11] corresponding to the basis set of choice. Though individual barrier heights may vary by 1-2 kcal/mol with the choice of method the overall trend is the same for all methods. See Supporting Information for full results.

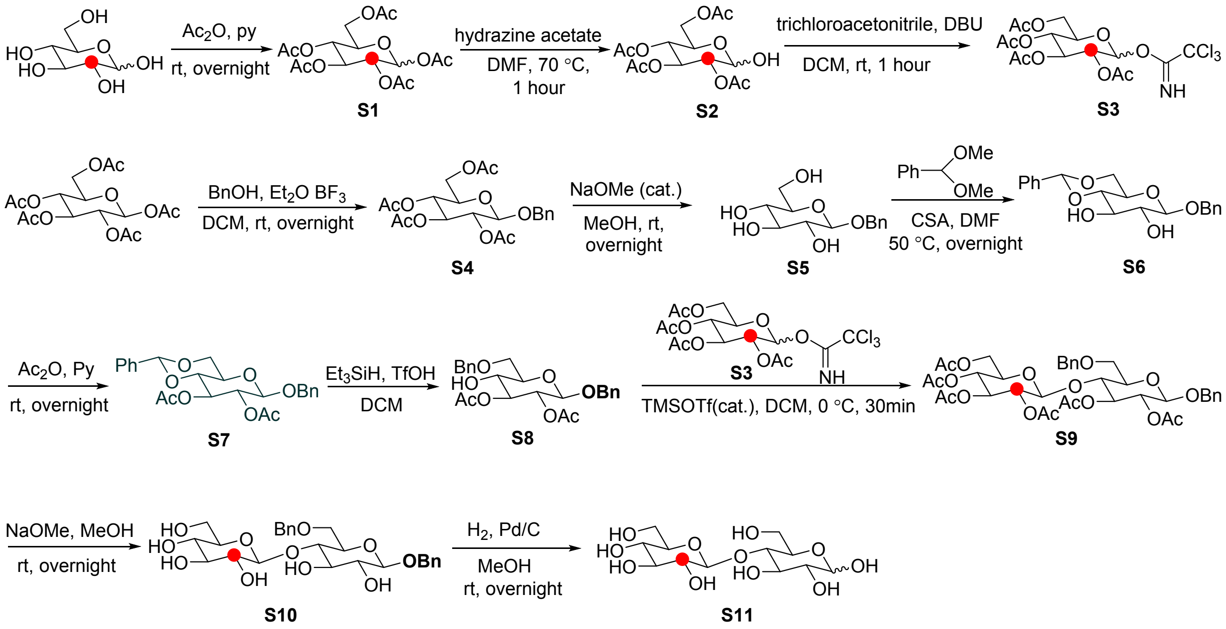

Scheme S1. Synthesis of ^{13}C-labeled cellobiose.

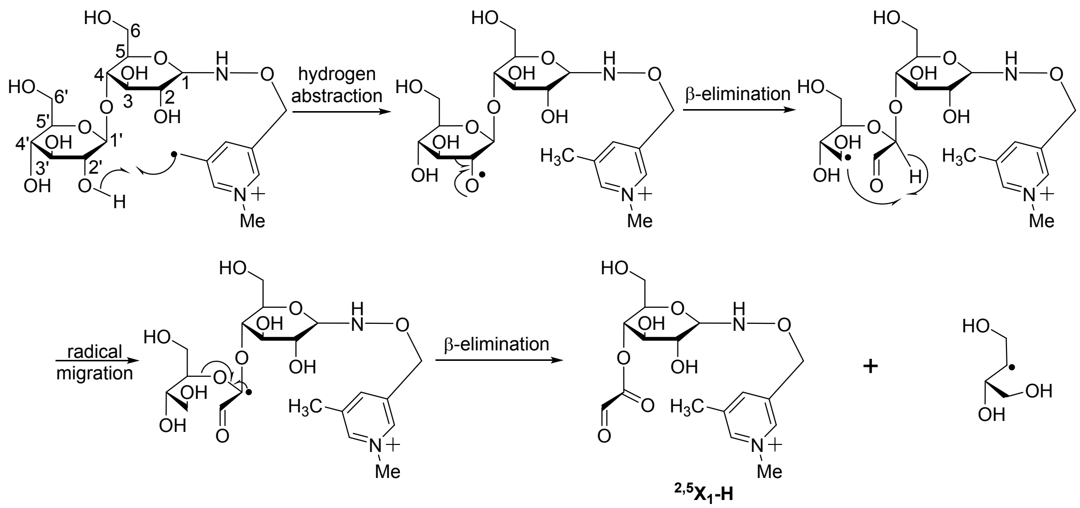

$^{2,5}X_1$-H

Scheme S2. The mechanism for the formation of $^{2,5}X_1$-H ion.

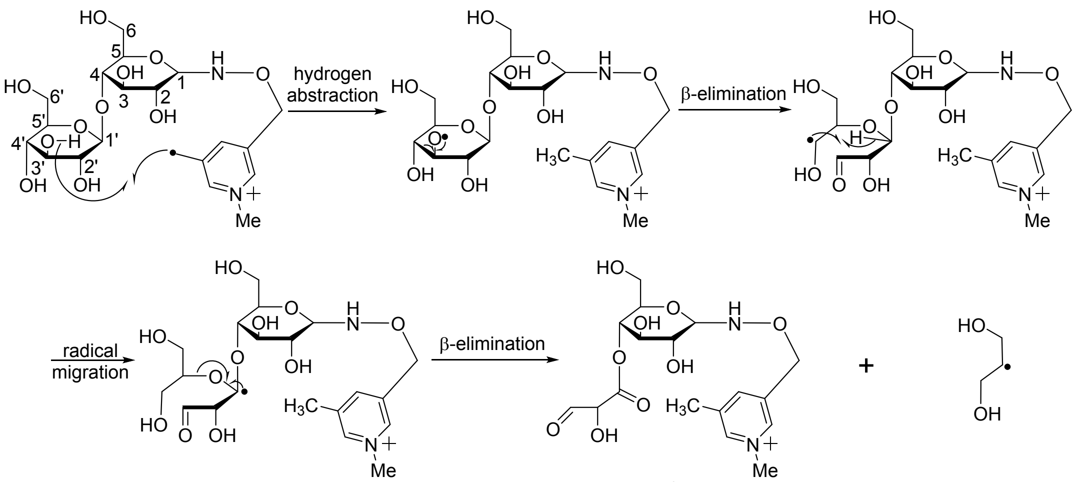

$^{3,5}X_1$-H

Scheme S3. The mechanism for the formation of $^{3,5}X_1$-H ion.

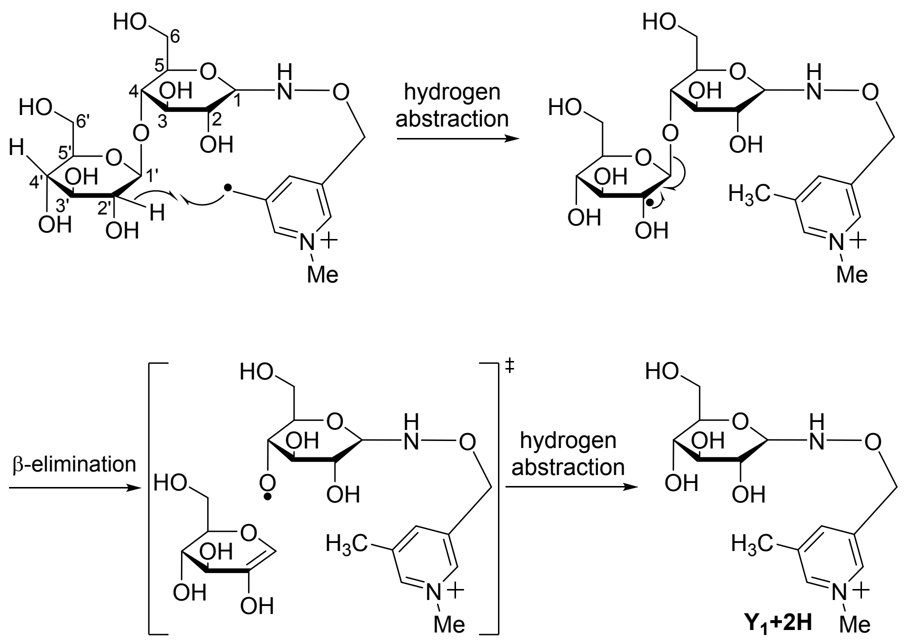

Scheme S4. The mechanism for the formation of Y_1+2H ion.

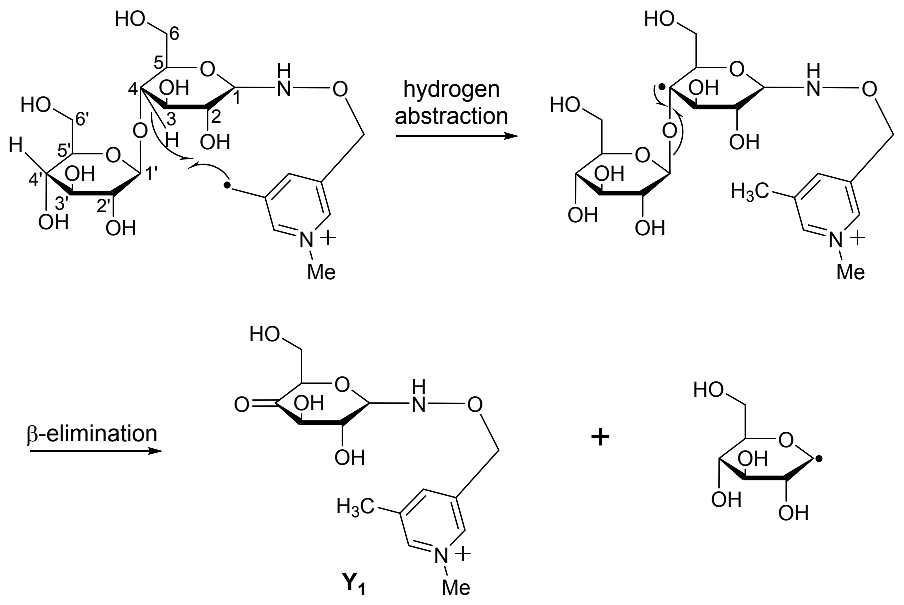

Scheme S5. The mechanism for the formation of Y_1 ion.

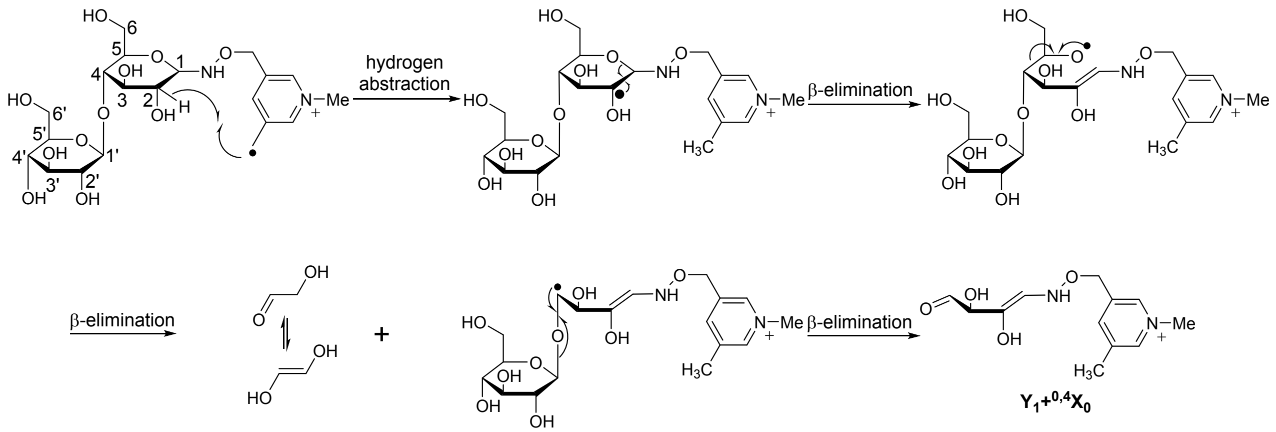

Scheme S6. The mechanism for the formation of Y_1+$^{0,4}X_0$ ion.

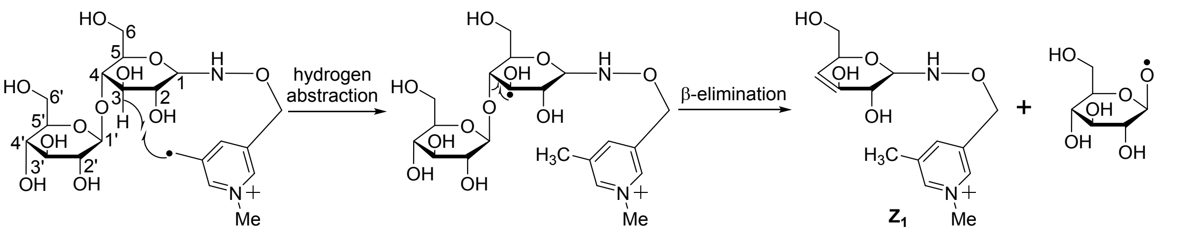

Scheme S7. The mechanism for the formation of Z_1 ion.

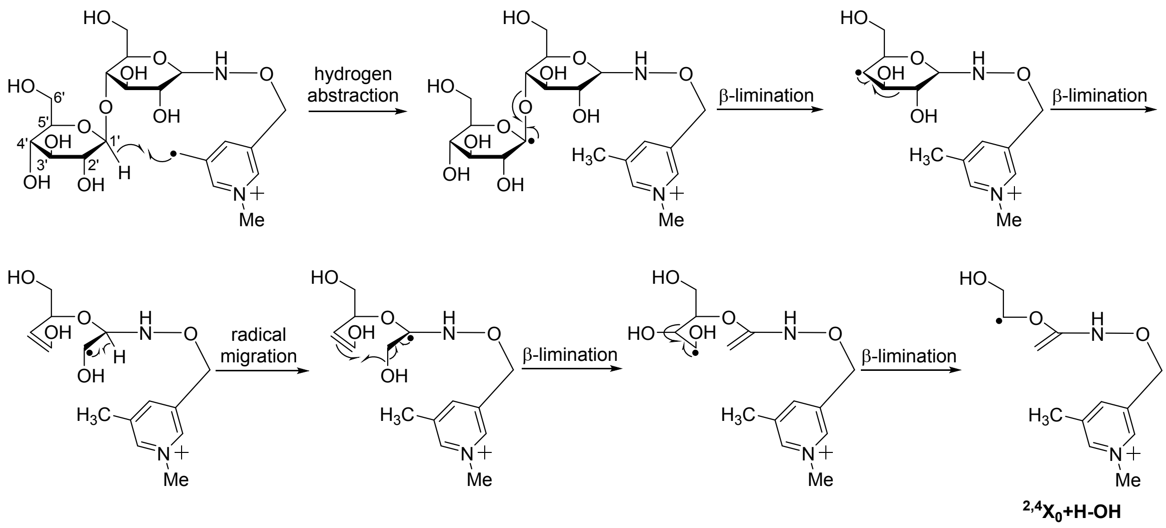

Scheme S8. Mechanism for the formation of $^{2,4}X_0$+H-OH ion.

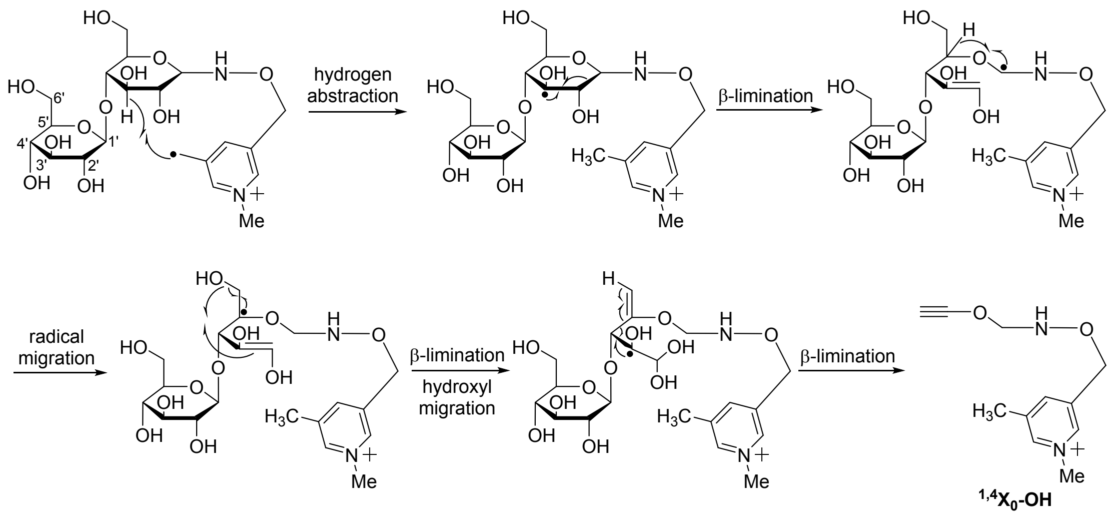

Scheme S9. Mechanism for the formation of $^{1,4}X_0$-OH ion.

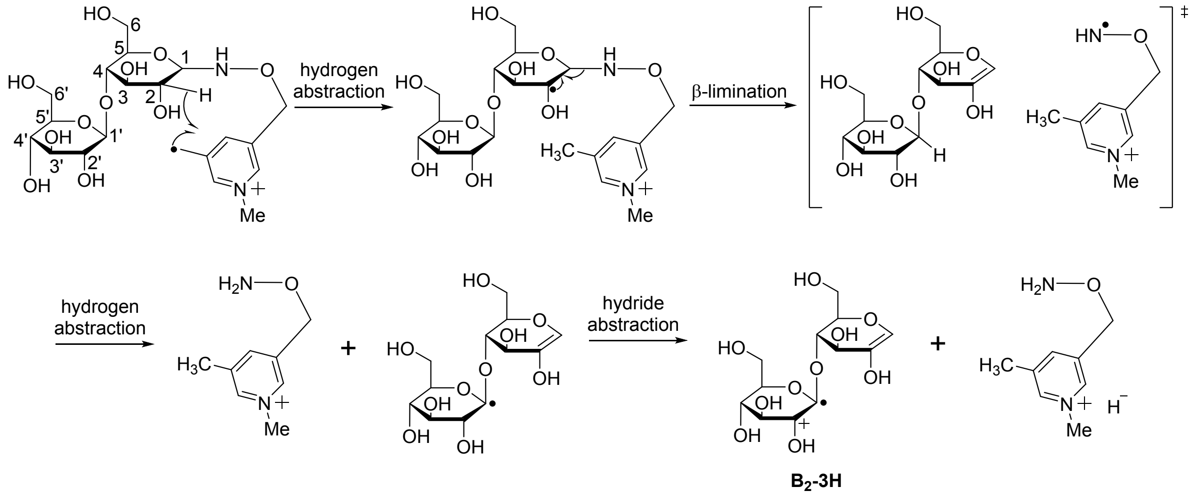

Scheme S10. The mechanism for the formation of B_2-3H ion.

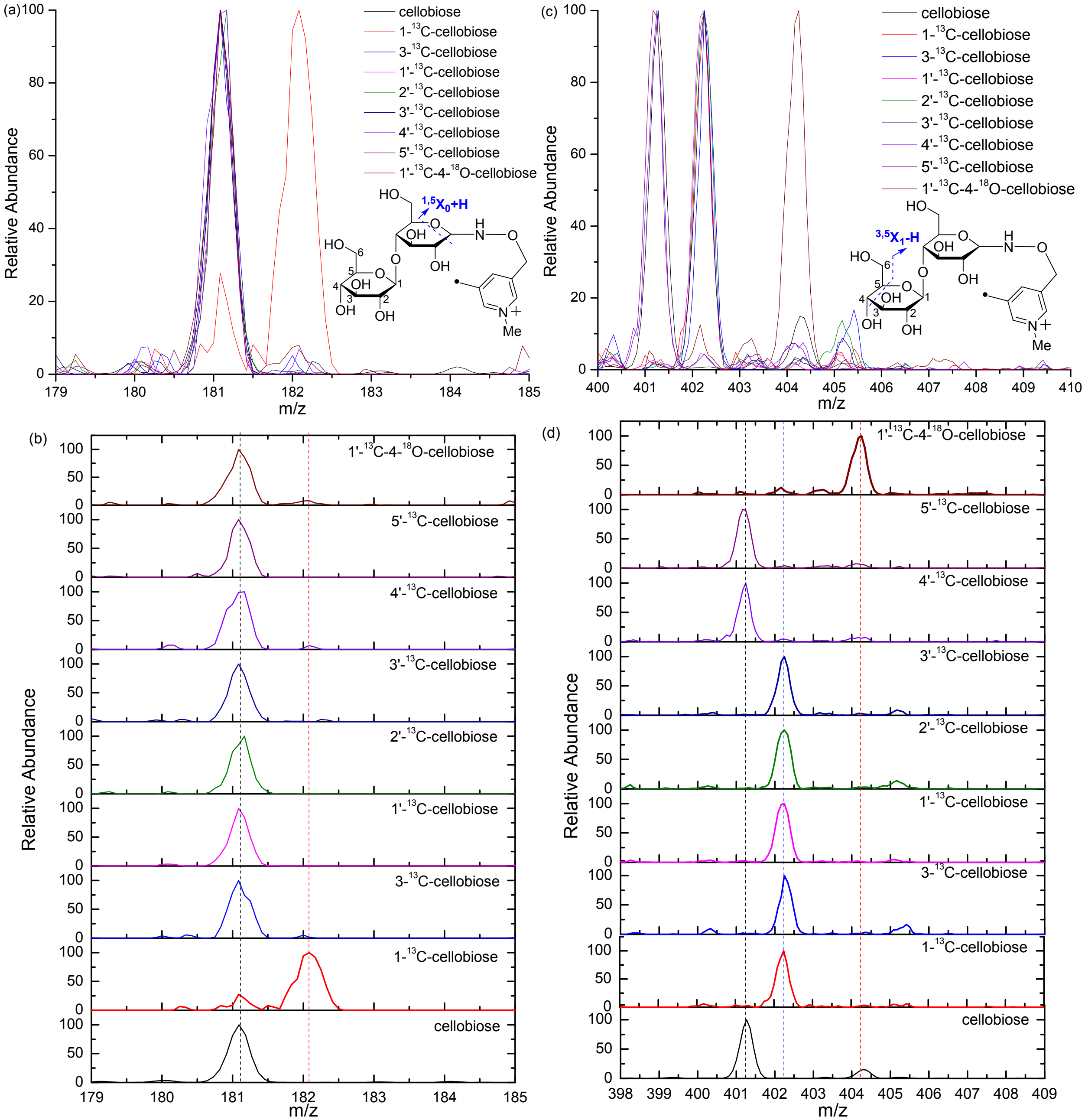

Figure S1. Zoom-in views (a, $^{1,5}X_0$+H), Stack views (b, $^{1,5}X_0$+H), zoom-in views (c, 400-409, $^{3,5}X_1$-H), and stack views (d, 399-409, $^{3,5}X_1$-H) of CID spectra of the seven Me-FRAGS derivatized $^{13}C/^{18}O$ labeled cellobioses and Me-FRAGS derivatized unlabeled cellobiose.

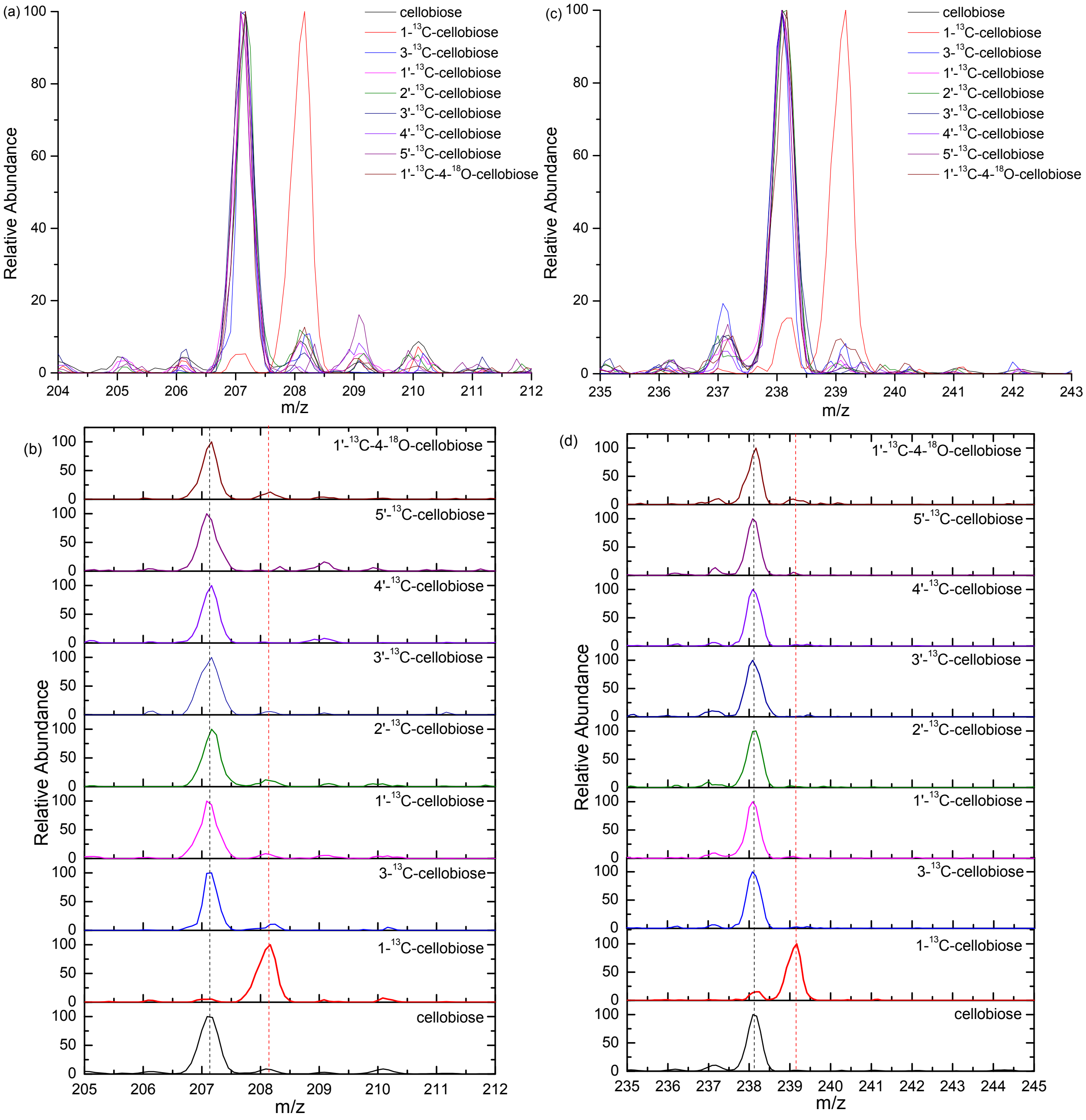

Figure S2. Zoom-in views (a, 204-212, $^{1,4}X_0$-OH), stack views (b, 205-212, $^{1,4}X_0$-OH), zoom-in views (c, 235-243, $^{2,4}X_0$+H-OH), and stack views (d, 235-245, $^{2,4}X_0$+H-OH) of CID spectra of the seven Me-FRAGS derivatized $^{13}C/^{18}O$ labeled cellobioses and Me-FRAGS derivatized unlabeled cellobiose.

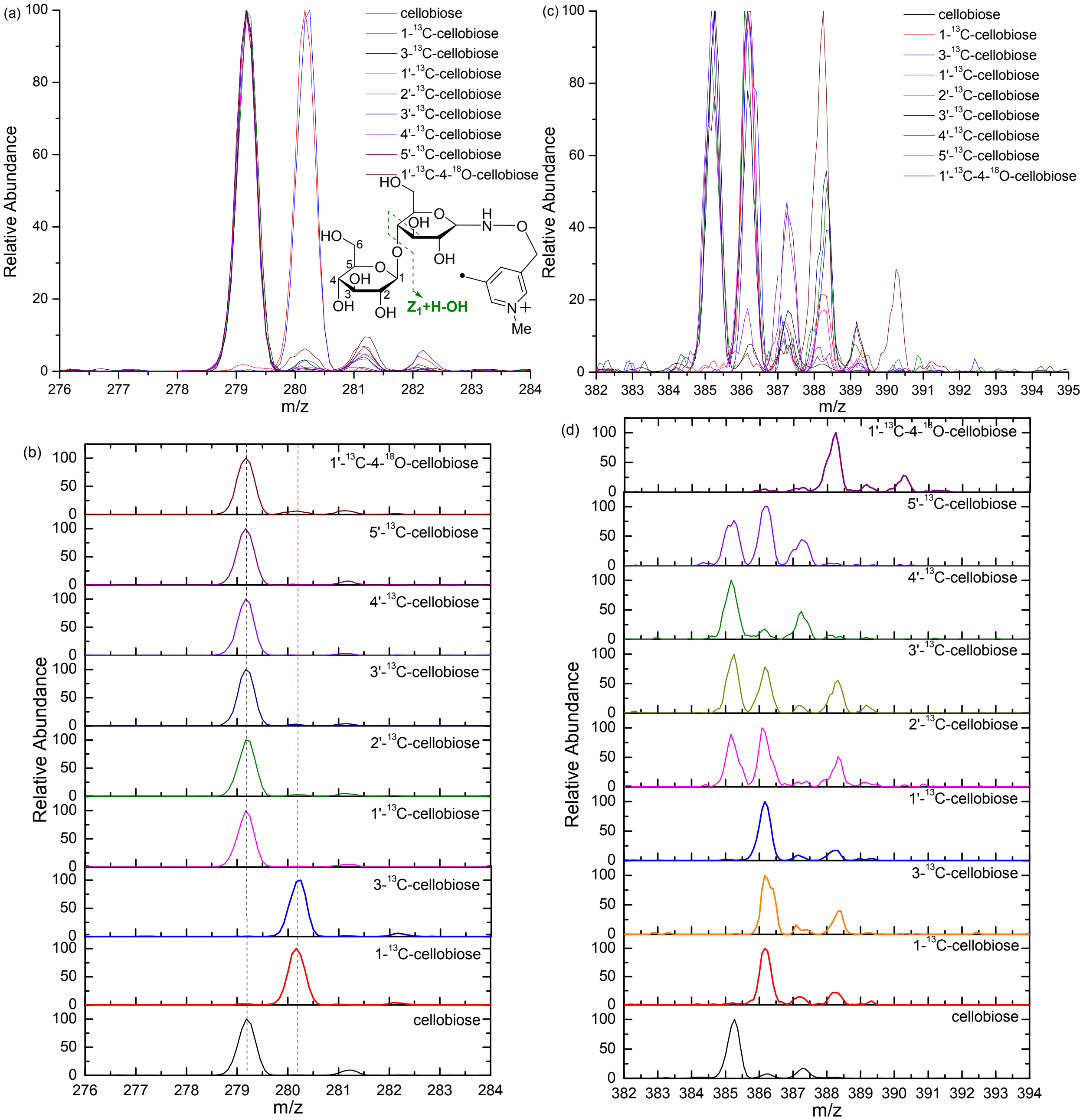

Figure S3. Zoom-in views (a, Z_1+H-OH), Stack views (b, Z_1+H-OH), zoom-in views (c, $^{0,3}X_1$-H or $^{1,4}X_1$-H), and stack views (d, $^{0,3}X_1$-H or $^{1,4}X_1$-H) of CID spectra of the seven Me-FRAGS derivatized $^{13}C/^{18}O$ labeled cellobioses and Me-FRAGS derivatized unlabeled cellobiose.

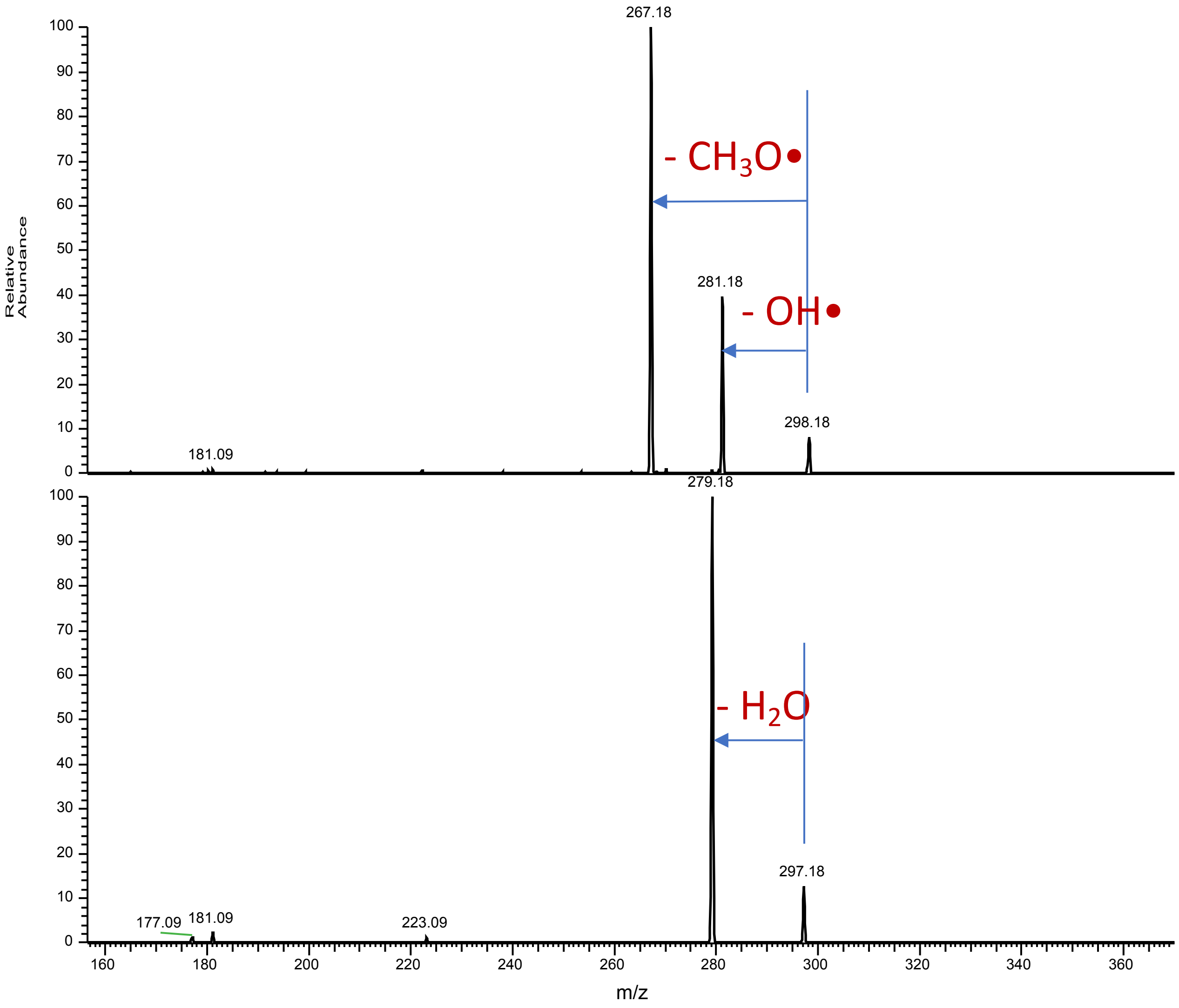

Figure S4. CID spectra of Z_1+H (m/z 298) and Z_1 (m/z 297) ions of cellobiose.

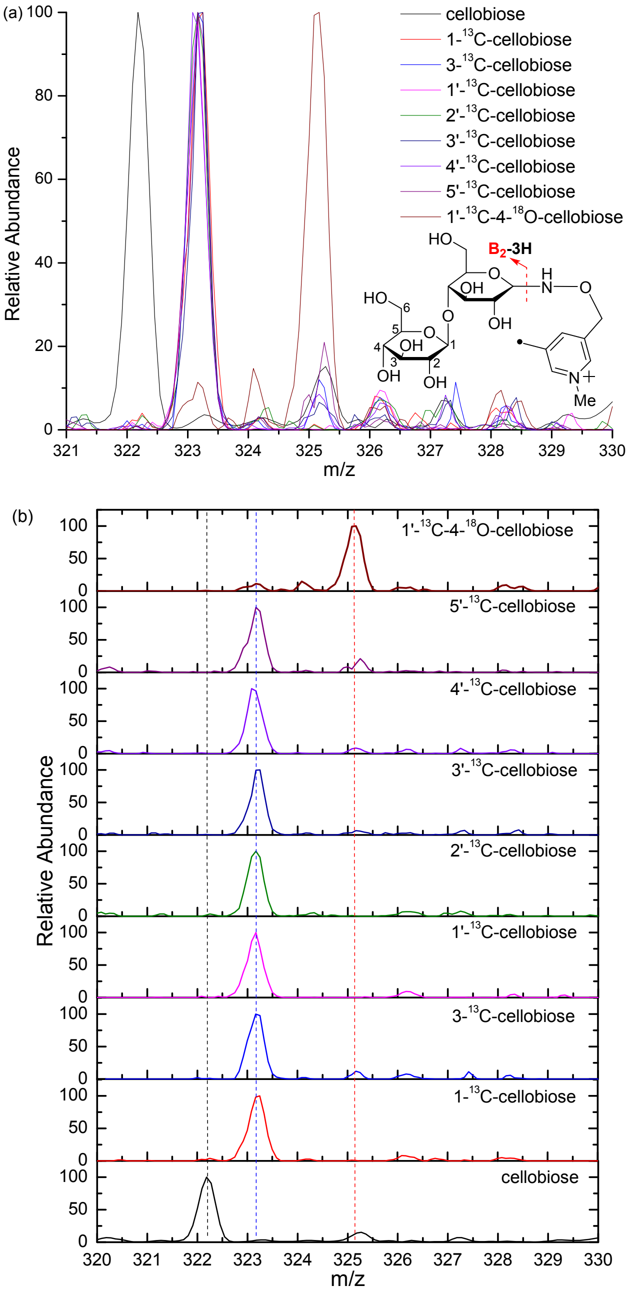

Figure S5. Zoom-in views (a, 321-330, B_2-3H) and stack views (b, 320-330, B_2-3H) of CID spectra of the seven Me-FRAGS derivatized $^{13}C/^{18}O$ labeled cellobioses and Me-FRAGS derivatized unlabeled cellobiose.

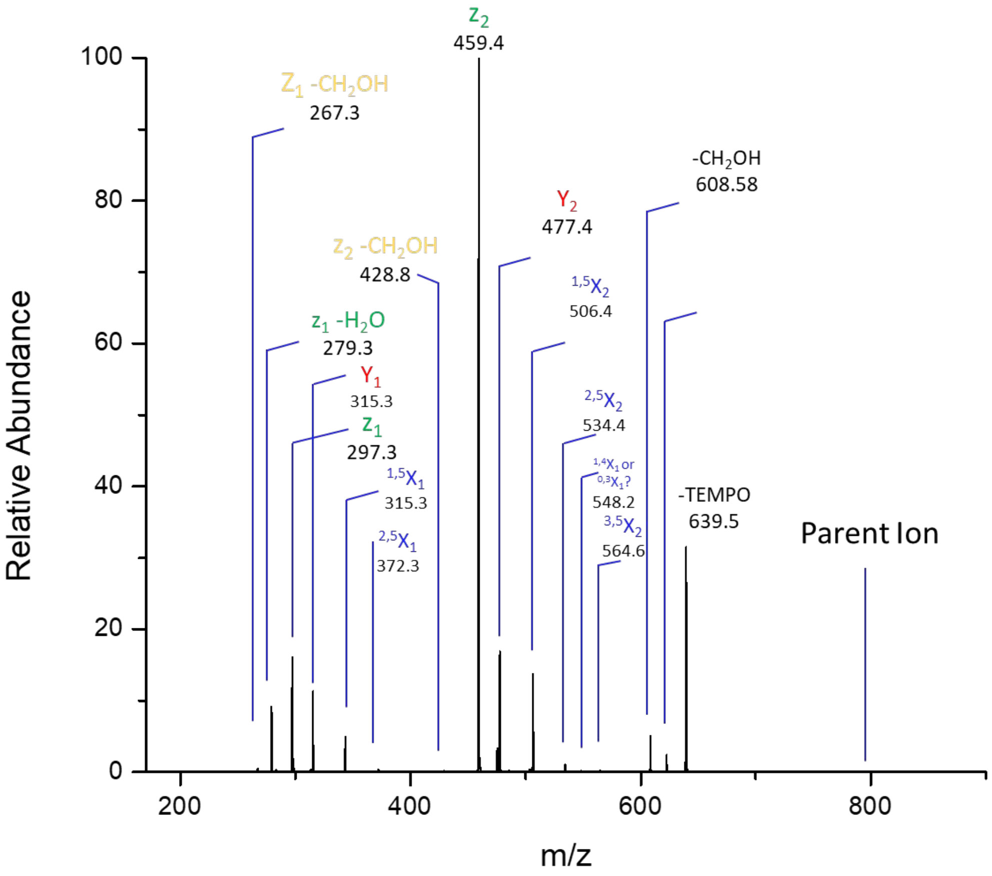

Figure S6. CID spectrum of Me-FRAGS derivatized 1-^{13}C-cellotriose.

Synthesis procedures

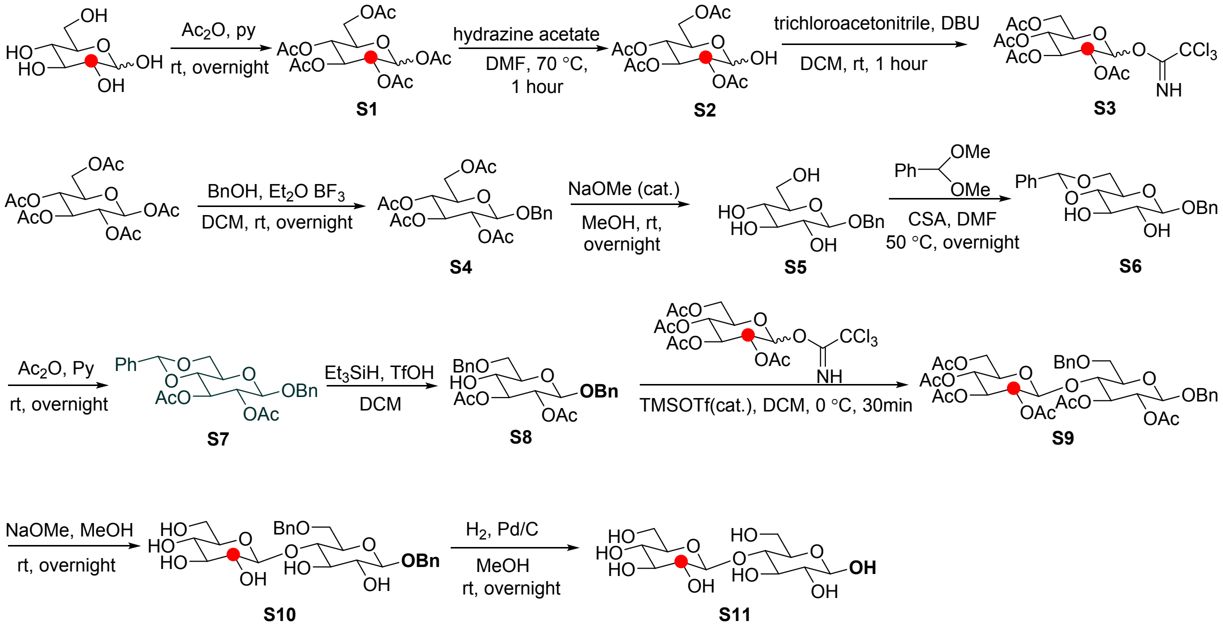

Scheme S1. Synthesis of [2'-^{13}C]-cellobiose.

General

The synthesis of ^{13}C-labeled cellobioses are achieved by following the procedures reported in literatures.[12-15] The synthesized compounds were purified by column chromatography on a Teledyne-ISCO CombiFlash Rf+. The compounds were characterized by ^{1}H-NMR and ^{13}C-NMR. $CDCl_3$, CD_3OD and D_2O were used as the NMR solvent. Compound **S3** and **S8** was synthesized using literature method.[14] **S3:** ^{1}H NMR (300 Hz, $CDCl_3$, δ), 8.69 (1H, s, NH), 6.56 (1H, d, J=3Hz, CH, C1), 6.15-4.16 (6H, m, CH, CH_2), 2.08 (3H, s, C=OCH_3), 2.05 (3H, s, C=OCH_3), 2.03 (3H, s, C=OCH_3), 2.02 (3H, s, C=OCH_3); ^{13}C NMR (75 Hz, $CDCl_3$, δ): 170.40, 169.86, 169.71, 169.39, 169.36, 160.59, 160.56, 93.03, 92.73, 92.42, 90.52, 73.30, 69.84, 69.55, 69.23, 67.63, 67.58, 61.22. **S8**: ^{1}H NMR (300 Hz, $CDCl_3$, δ), 7.28-7.18 (10H, m), 4.97-4.88 (2H, m), 4.80 (1H, d, J=12Hz), 4.57-4.50 (3H, m), 4.47-4.43 (1H, m), 3.76-3.65 (3H, m), 3.47-3.40 (1H, m), 2.93 (1H, d, J=3Hz), 1.99 (3H, s), 1.92 (3H, s); ^{13}C NMR (75 Hz, $CDCl_3$, δ): 171.28, 169.65, 137.53, 136.94, 128.49, 128.38, 127.90, 127.86, 127.74, 127.64, 99.43, 75.67, 74.13, 73.78, 71.32, 70.81, 70.64, 70.09.

benzyl 2,3,4,6-tetra-O-acetyl-2-^{13}C-β-D-glucopyranosyl-(1-4)-2,3-di-O-acetyl-6-O-benzyl-β-D-glucopyranoside (S9)

A mixture of **S3** (0.050 g, 0.11 mmol), **S8** (0.066 g, 0.13 mmol), 4 Å molecular sieves in anhydrous DCM was stirred at room temperature under argon for 30 minutes. Then this mixture was cooled –20 ℃, TMSOTf (5.7 μL, 0.032 mmol) was added slowly over 30 minutes. The reaction was warmed slowly room temperature in 1 hour. The reaction was quenched by the addition of 0.30 mL triethylamine and filtered. The filtrate was concentrated and purified by flash chromatography (ethyl acetate:heane = 1:1) to give **S9** (0.055 g, 64%). ^{1}H NMR (400 Hz, $CDCl_3$, δ): 7.44-7.26 (10H, m, Ar-H), 5.11-3.94 (14H, m, CH, *Ph*CH_2), 3.75-3.38 (4H, m, CH_2); ^{13}C NMR (100 Hz, $CDCl_3$, δ): 170.50, 170.24, 170.01, 169.57, 169.32, 168.75, 137.67, 136.93, 128.66, 128.40, 128.15, 128.06, 127.89, 100.21, 99.97, 99.73, 99.50, 75.24, 74.97, 74.94, 74.68, 73.70, 73.27, 72.87, 72.58, 71.82, 71.75, 71.58, 70.99, 70.66, 67.87, 67.13, 61.54. MS (ESI) Calcd. for $^{13}CC_{37}H_{46}O_{17}Na$: 798.26 $[M+Na]^+$, found: 798.19.

benzyl 2-^{13}C-β-D-glucopyranosyl-(1-4)-6-O-benzyl-β-D-glucopyranoside (S10)

The deacetylation of trisaccharide **S9** was achieved under Zempler conditions.[14] To a solution of **S9** (0.055 g, 0.070 mmol) in 5 mL anhydrous methanol NaOMe was added to adjust the pH to 9. The reaction mixture was stirred overnight, quenched by Amberlyst 15 acid resin, concentrated, and purified by flash chromatography (methanol:dichloromethane = 1:5) to afford **S10** in quantitative yield. ^{1}H NMR (400 Hz, methanol-d_4, δ): 7.41-7.22 (10H, m, Ar-H), 4.84 (1H, d, overlap with solvent peak), 4.64 (1H, d, J= 16Hz), 4.57 (2H, d, 4Hz), 4.34 (2H, t, 9Hz), 3.94-3.81 (3H, m), 3.66-3.59 (2H, m), 3.54-3.41 (3H, m), 3.34-3.31 (2H, m), 3.27-3.23 (2H, m); ^{13}C NMR (100 Hz, methanol-d_4, δ): 139.85, 139.14, 129.54, 129.42, 129.33, 129.09, 128.85, 128.82, 105.04, 104.73, 104.42, 103.41, 80.69, 80.65, 80.19, 78.30, 78.25, 77.78, 76.51, 75.90, 75.37, 75.30, 75.07, 74.78, 74.75, 74.57, 74.37, 72.08, 71.52, 71.49, 69.93, 62.59. MS (ESI) Calcd. for $^{13}CC_{25}H_{34}O_{11}Na$: 546.20 $[M+Na]^+$, found: 546.18

2-^{13}C-β-D-glucopyranosyl-(1-4)-β-D-glucopyranoside (S11)

The above intermediate **S10** was dissolved in 5 mL of methanol/water (1:1), and a catalytic amount of palladium on activated charcoal (1 mg) was added into the solution. The reaction mixture was stirred overnight under a two layered balloon was filled with H_2 gas. The reaction mixture was

filtered and the filtrate was concentrated and purified by flash chromatography to give **S11,** [2'-^{13}C]-cellobiose (0.0215 g, 90%). ^{1}H NMR (400 Hz, D_2O, δ): 4.60 (1H, d, J=12Hz, CH, C1'), 4.45(1H, d, J=12Hz, CH, C1), 3.95-3.11 (12H, m, CH, CH2, ring CH); ^{13}C NMR (100 Hz, D_2O, δ): 102.88, 102.54, 102.23, 95.78, 91.84, 76.01, 75.29, 74.80, 74.31, 73.93, 73.20, 71.78, 71.69, 70.13, 69.51, 60.61, 60.01. MS (ESI) Calcd. for $^{13}CC_{11}H_{22}O_{11}Na$: 366.11 $[M+Na]^+$, found: 366.12

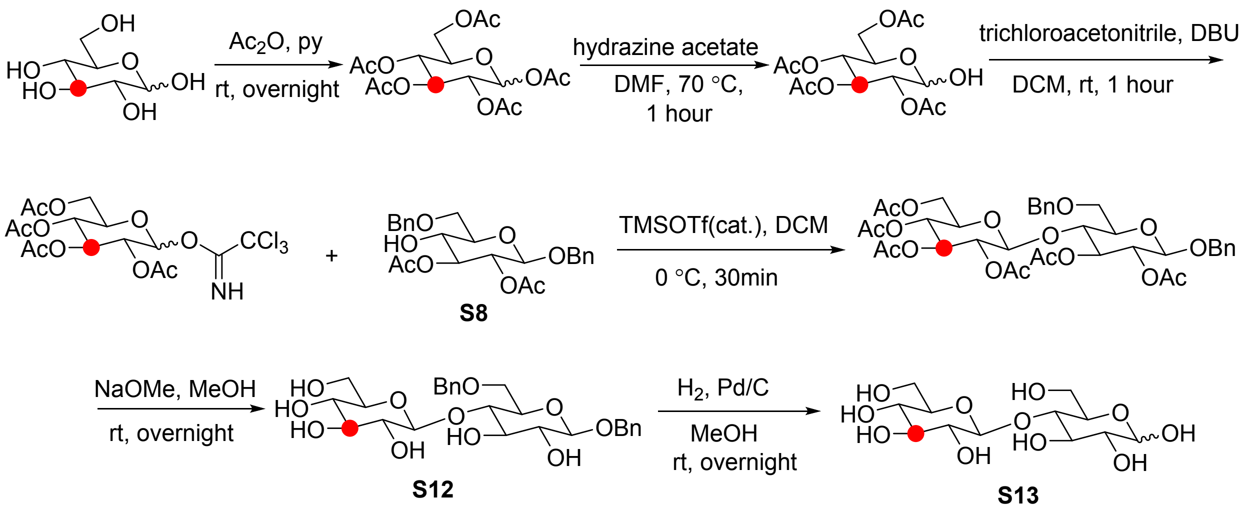

Scheme S2. Synthesis of [3'-^{13}C]-cellobiose.

Same synthesis strategy was employed for the synthesis of [3'-^{13}C]-cellobiose.

The intermediate **S12** was dissolved in 5 mL of methanol/water (1:1), and a catalytic amount of palladium on activated charcoal (1 mg) was added into the solution. The reaction mixture was stirred overnight under a two layered balloon was filled with H_2 gas. The reaction mixture was filtered and the filtrate was concentrated and purified by flash chromatography to give **S13,** [3'-^{13}C]-cellobiose. ^{1}H NMR (400 Hz, D_2O, δ): 4.60 (1H, d, J=12Hz, CH, C1'), 4.45(1H, d, J=12Hz, CH, C1), 3.93-3.14 (12H, m, CH, CH2, ring CH); ^{13}C NMR (100 Hz, D_2O, δ): 105.43, 105.40, 105.37, 105.34, 98.95, 94.66, 81.62, 81.49, 78.85, 78.82, 78.62, 78.35, 78.11, 77.63, 77.14, 76.74, 76.24, 75.72, 75.70, 74.18, 74.07, 72.95, 72.55, 72.03, 71.24, 63.46, 63.41, 62.92, 62.79. MS (ESI) Calcd. for $^{13}CC_{11}H_{22}O_{11}Na$: 366.11 $[M+Na]^+$, found: 366.10

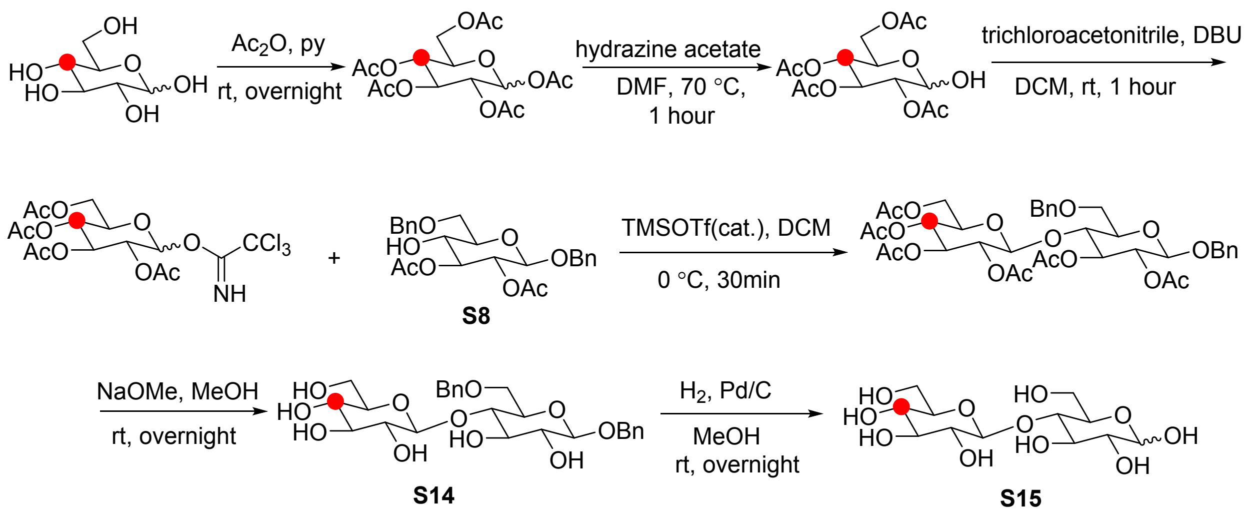

Scheme S3. Synthesis of [4’-^{13}C]-cellobiose.

Same synthesis strategy was employed for the synthesis of [4’-^{13}C]-cellobiose.

The intermediate **S14** was dissolved in 5 mL of methanol/water (1:1), and a catalytic amount of palladium on activated charcoal (1 mg) was added into the solution. The reaction mixture was stirred overnight under a two layered balloon was filled with H_2 gas. The reaction mixture was filtered and the filtrate was concentrated and purified by flash chromatography to give **S15,** [4’-^{13}C]-cellobiose. ^{1}H NMR (400 Hz, D_2O, δ): 4.61 (1H, d, J=8Hz, CH, C1’), 4.46 (1H, d, J=8Hz, CH, C1), 3.95-3.11 (12H, m, CH, CH2, ring CH); ^{13}C NMR (100 Hz, D_2O, δ): 105.41, 98.60, 94.67, 81.60, 81.46, 78.35, 77.65, 77.15, 76.74, 75.26, 74.18, 74.08, 73.66, 73.28, 72.97, 72.31, 72.18, 71.86, 70.97, 70.12, 69.72, 68.99, 67.67, 63.43, 62.90, 62.77. MS (ESI) Calcd. for $^{13}CC_{11}H_{22}O_{11}Na$: 366.11 $[M+Na]^+$, found: 366.14

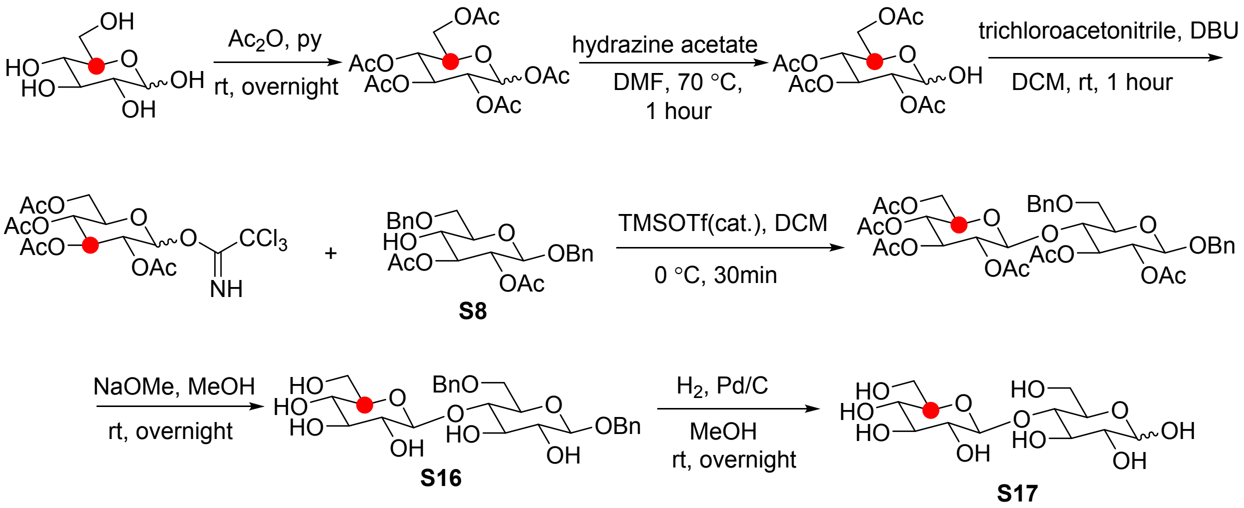

Scheme S4. Synthesis of [5’-^{13}C]-cellobiose.

Same synthesis strategy was employed for the synthesis of [5’-^{13}C]-cellobiose.

The intermediate **S16** was dissolved in 5 mL of methanol/water (1:1), and a catalytic amount of palladium on activated charcoal (1 mg) was added into the solution. The reaction mixture was stirred overnight under a two layered balloon was filled with H_2 gas. The reaction mixture was filtered and the filtrate was concentrated and purified by flash chromatography to give **S17,** [5’-^{13}C]-cellobiose. ^{1}H NMR (400 Hz, D_2O, δ): 4.63 (1H, d, J=8Hz, CH, C1’), 4.48 (1H, d, J=8Hz, CH, C1), 4.01-3.23 (12H, m, CH, CH2, ring CH); ^{13}C NMR (100 Hz, D_2O, δ): 105.40, 98.61, 94.66, 85.72, 84.85, 83.34, 83.15, 82.25, 81.60, 81.46, 81.36, 80.60, 80.37, 78.83, 78.34, 77.63, 77.13, 76.98, 76.91, 76.75, 76.01, 75.89, 75.53, 74.32, 74.17, 74.08, 72.95, 72.49, 72.08, 63.62, 63.42, 63.20, 62.90, 62.76. MS (ESI) Calcd. for $^{13}CC_{11}H_{22}O_{11}Na$: 366.11 $[M+Na]^+$, found: 366.09

Computational Results

Scheme	Ion	PW6B95-D3	PBE0-D3	RPA
2	$^{1,5}X_1$+H	0	0	0
3	$^{2,5}X_1$-H	5.9	7.2	8.6
4	$^{3,5}X_1$-H	8.3	9.5	10.3
5	Y_1+2H	4.9	4.6	5.5
6	Y_1	17.5	18.8	18.9
7	$Y_1+{}^{0,4}X_0$	9.3	10.3	10.3
8	Z_1	4.7	4.1	5.2
9	Z_1+H	13.5	15	14.4

Table 1: Relative electronic energies (in kcal/mol) for the transition states for the sequential hydrogen abstraction for the different fragmentation schemes presented in **Scheme 2-9**. The energies were obtained with the PBE0 functional and the PW6B95 functional both with Grimme's D3-BJ dispersion correction using the def2-TZVPP basis set. The RPA results were obtained using self-consistent PBE orbitals and the def2-QZVPP basis set. See the Computational Methods section for full details on the calculations and how transition state structures were obtained.

References

1. Pracht, P.; Bohle, F.; Grimme, S., Automated exploration of the low-energy chemical space with fast quantum chemical methods. *Phys. Chem. Chem. Phys.* **2020,** *22*, 7169-7192.

2. Perdew, J. P. B., Kieron; Ernzerhof, Matthias, Generalized gradient approximation made simple. *Phys. Rev. Lett.* **1996,** *77*, 3865-3868.
3. Weigend, F.; Ahlrichs, R., Balanced basis sets of split valence, triple zeta valence and quadruple zeta valence quality for H to Rn: Design and assessment of accuracy. *Phys. Chem. Chem. Phys.* **2005,** *7*, 3297-3305.
4. Adamo, C. B., Vincenzo, Toward reliable density functional methods without adjustable parameters: The PBE0 model. *J. Chem. Phys.* **1999,** *110*, 6158-6170.
5. Zhao, Y.; Truhlar, D. G., Design of density functionals that are broadly accurate for thermochemistry, thermochemical kinetics, and nonbonded interactions. *J. Phys. Chem. A* **2005,** *109*, 5656-5667.
6. Becke, A. D.; Johnson, E. R., A density-functional model of the dispersion interaction. *J. Chem. Phys.* **2005,** *123*, 154101.
7. Grimme, S.; Antony, J.; Ehrlich, S.; Krieg, H., A consistent and accurate ab initio parametrization of density functional dispersion correction (DFT-D) for the 94 elements H-Pu. *J. Chem. Phys.* **2010,** *132* 154104.
8. Eshuis, H.; Furche, F., A Parameter-Free Density Functional That Works for Noncovalent Interactions. *J. Phys. Chem. Lett.* **2011,** *2*, 983-989.
9. Hesselmann, A.; Gorling, A., Random-phase approximation correlation methods for molecules and solids. *Mol. Phys.* **2011,** *109*, 2473-2500.
10. Chen, G. P.; Voora, V. K.; Agee, M. M.; Balasubramani, S. G.; Furche, F., Random-Phase Approximation Methods. *Annu. Rev. Phys. Chem.* **2017,** *68*, 421-445.
11. Weigend, F., Accurate Coulomb-fitting basis sets for H to Rn. *Phys. Chem. Chem. Phys.* **2006,** *8*, 1057-1065.

12 Wang, Z.; Chinoy, Z. S.; Ambre, S. G.; Peng, W.; McBride, R.; Vries, R. P. de; Glushka, J.; Paulson, J. C.; Boons, G.-J. A General Strategy for the Chemoenzymatic Synthesis of Asymmetrically Branched N-Glycans. *Science* **2013**, *341*, 379–383.

13 Autar, R.; Liskamp, R. M. J.; Pieters, R. J. A Facile Synthesis of the GalNAcβ1→4Gal Target Sequence of Respiratory Pathogens. *Carbohydr. Res.* **2005**, *340*, 2436–2442.

14 Reina, J. J.; Rojo, J. Synthesis of 2-Azidoethyl α-d-Mannopyranoside Orthogonally Protected and Selective Deprotections. *Tetrahedron Lett.* **2006**, *47*, 2475–2478.

15 Söderman, P.; Widmalm, G. Stereospecific Deuteration in the Synthesis of Methyl α-(4-2H)-Cellobioside. *J. Org. Chem.* **1999**, *64*, 4199–4200.

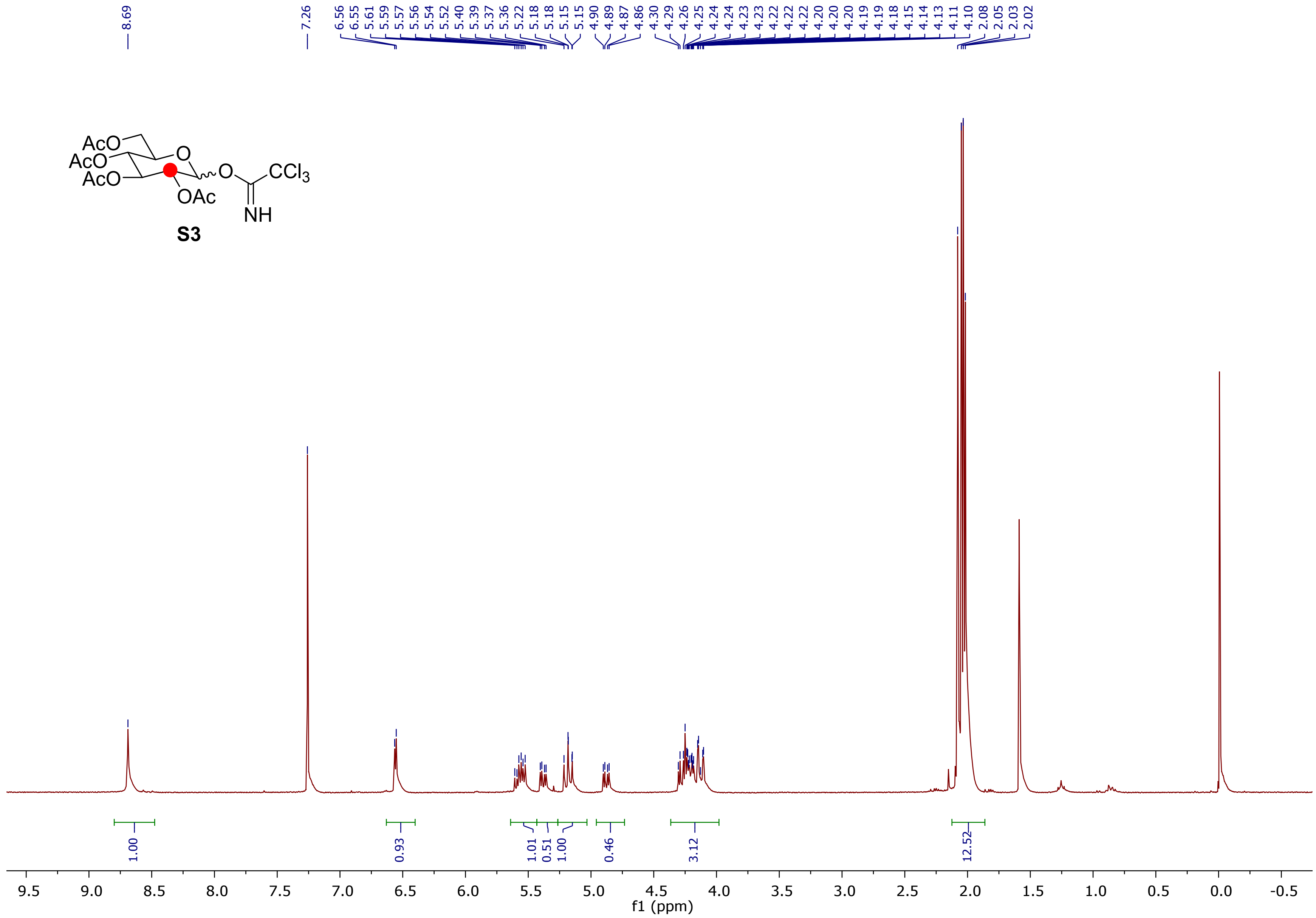
AcO
AcO
AcO
O
OAc
O
CCl3
NH
f1 (ppm)

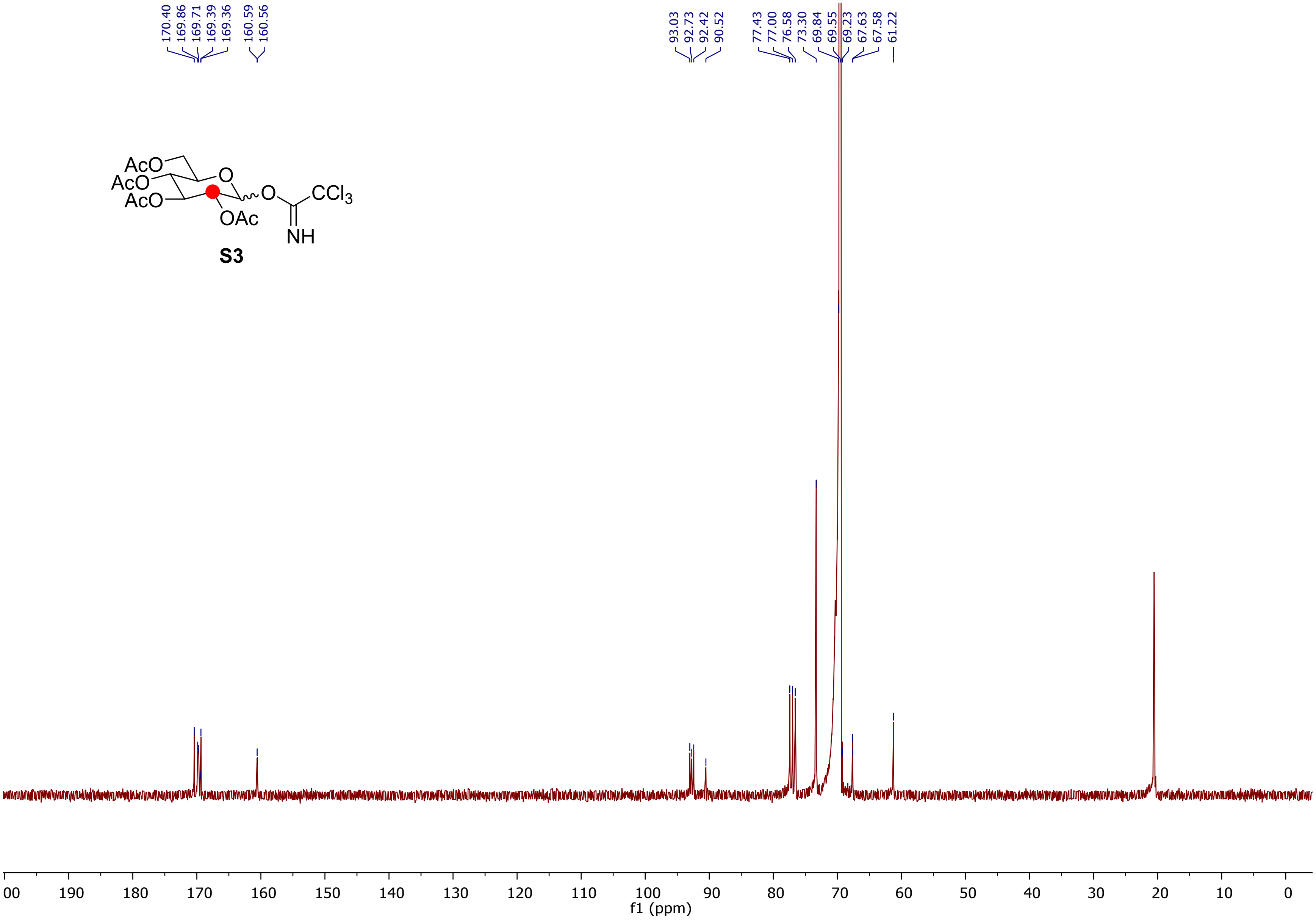
AcO
AcO
AcO
O
O
CCl3
OAc
NH
170.40
169.86
169.71
169.39
169.36
160.59
160.56
93.03
92.73
92.42
90.52
77.43
77.00
76.58
73.30
69.84
69.55
69.23
67.63
67.58
61.22
f1 (ppm)

S3

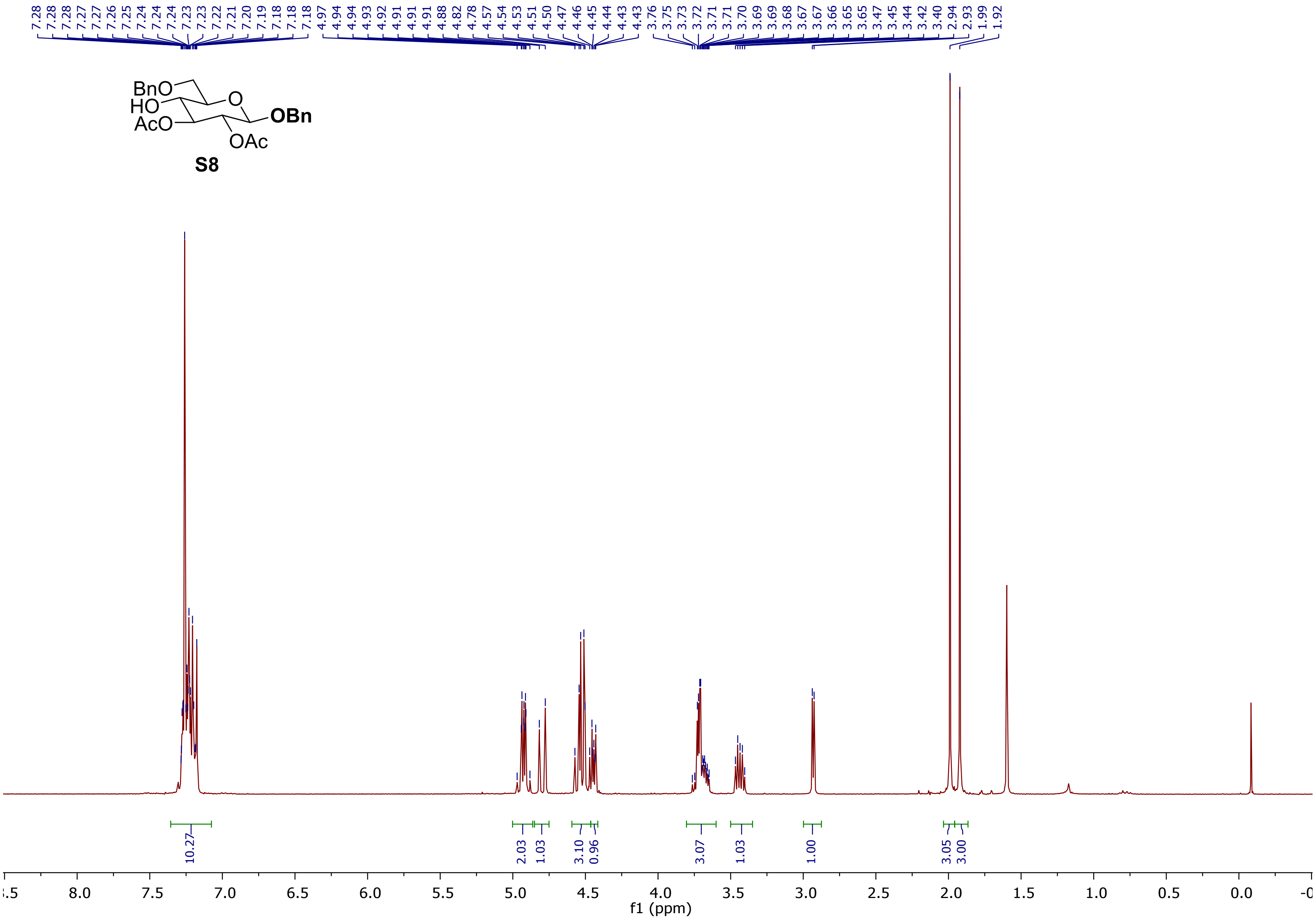

S8

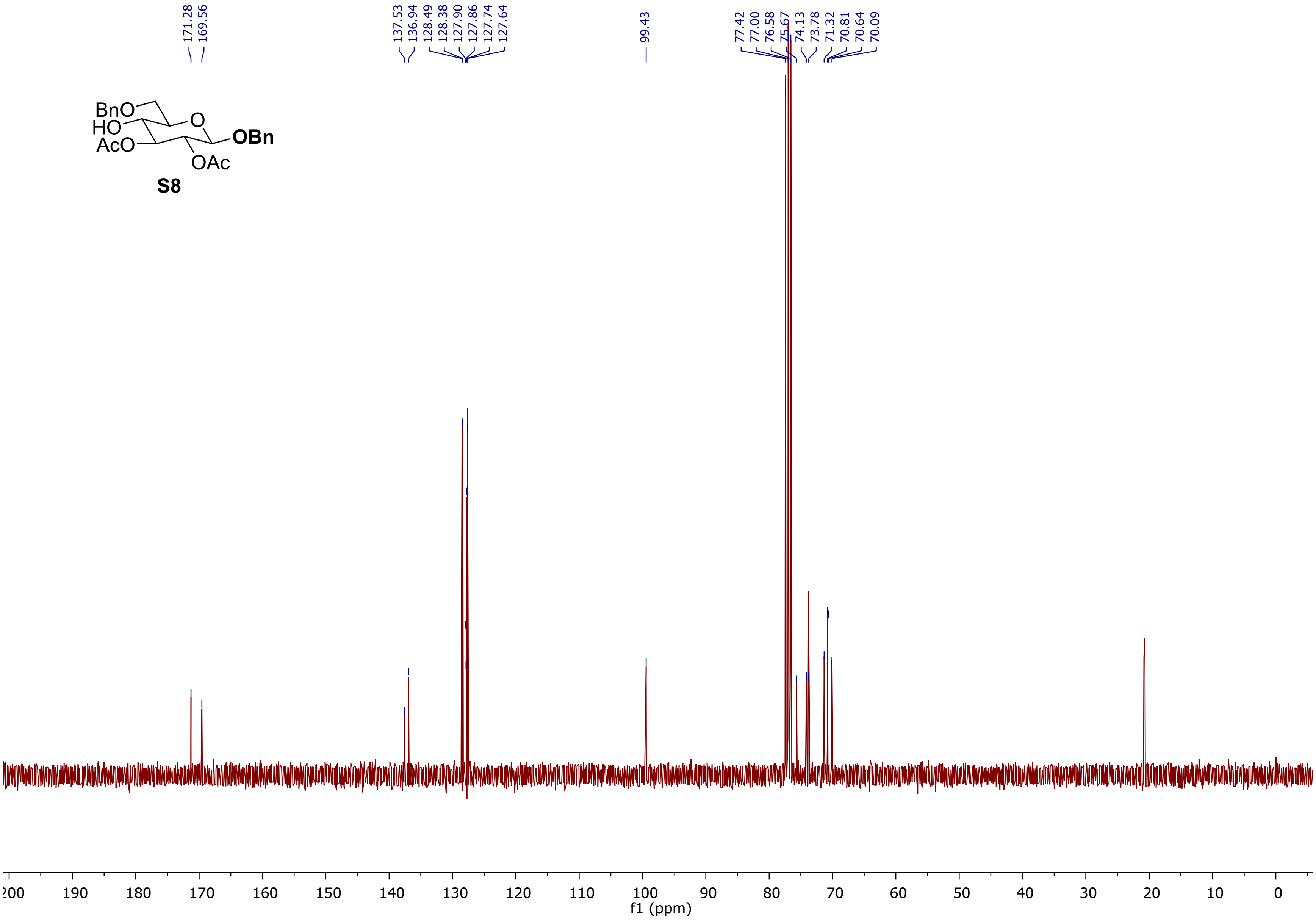
171.28
169.56
137.53
136.94
128.49
128.38
127.90
127.86
127.74
127.64
99.43
77.42
77.00
76.58
75.67
74.13
73.78
71.32
70.81
70.64
70.09
BnO
HO
AcO
O
OBn
OAc
S8
200 190 180 170 160 150 140 130 120 110 100 90 80 70 60 50 40 30 20 10 0
f1 (ppm)

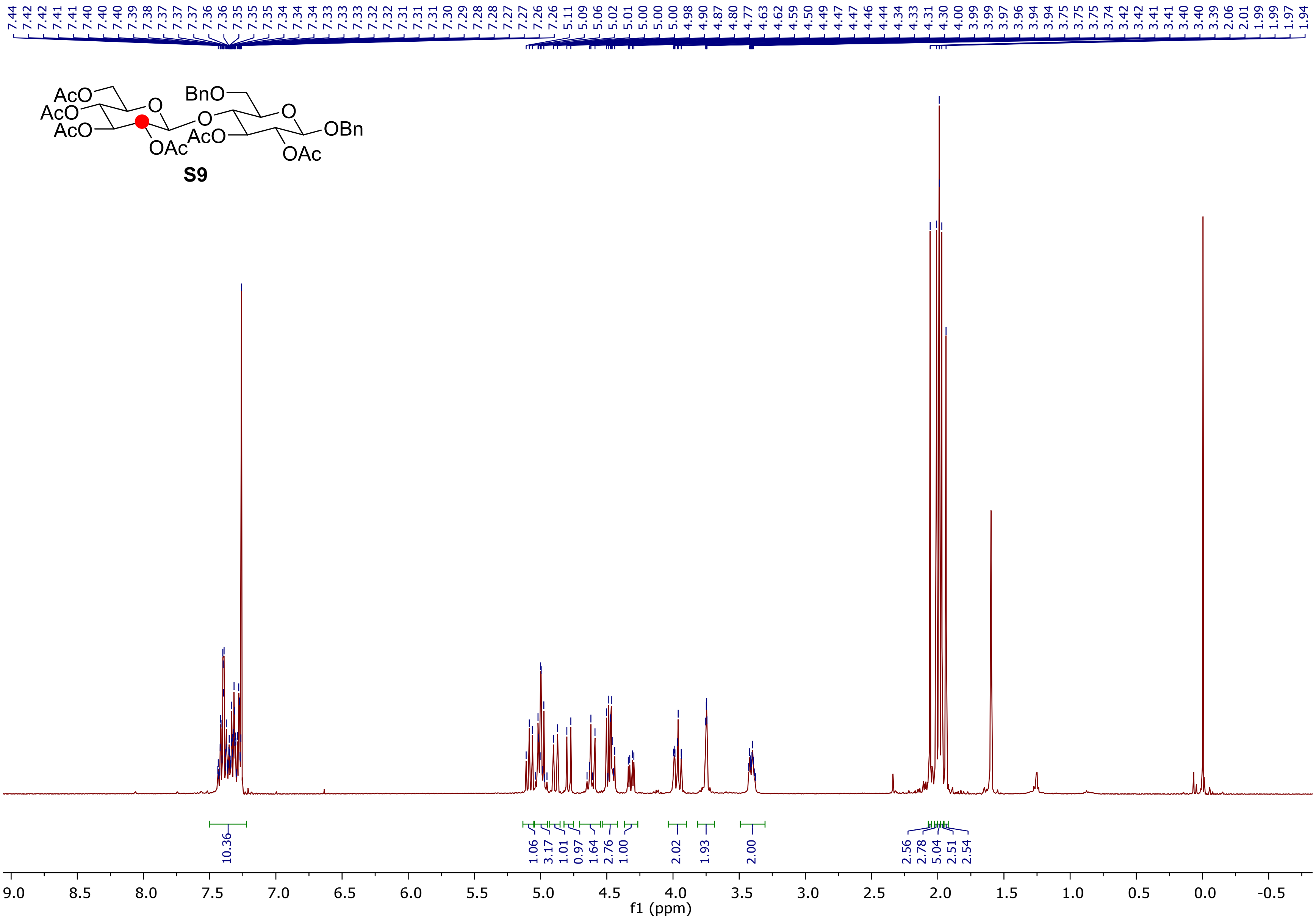

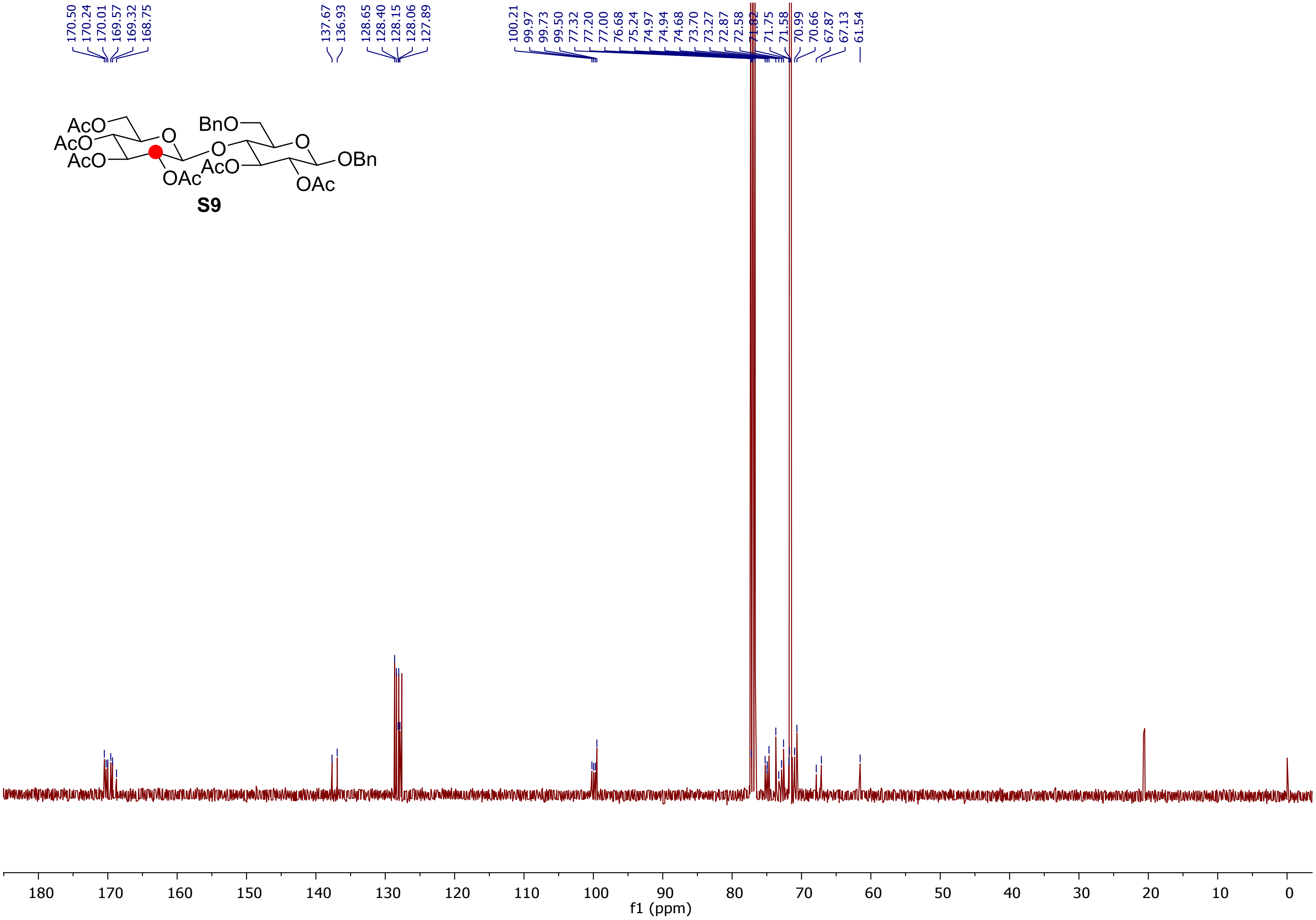
170.50
170.24
170.01
169.57
169.32
168.75
137.67
136.93
128.65
128.40
128.15
128.06
127.89
100.21
99.97
99.73
99.50
77.32
77.20
77.00
76.68
75.24
74.97
74.94
74.68
73.70
73.27
72.87
72.58
71.82
71.75
71.58
70.99
70.66
67.87
67.13
61.54
AcO
AcO
AcO
O
OAc
BnO
O
AcO
O
OBn
OAc
S9
f1 (ppm)

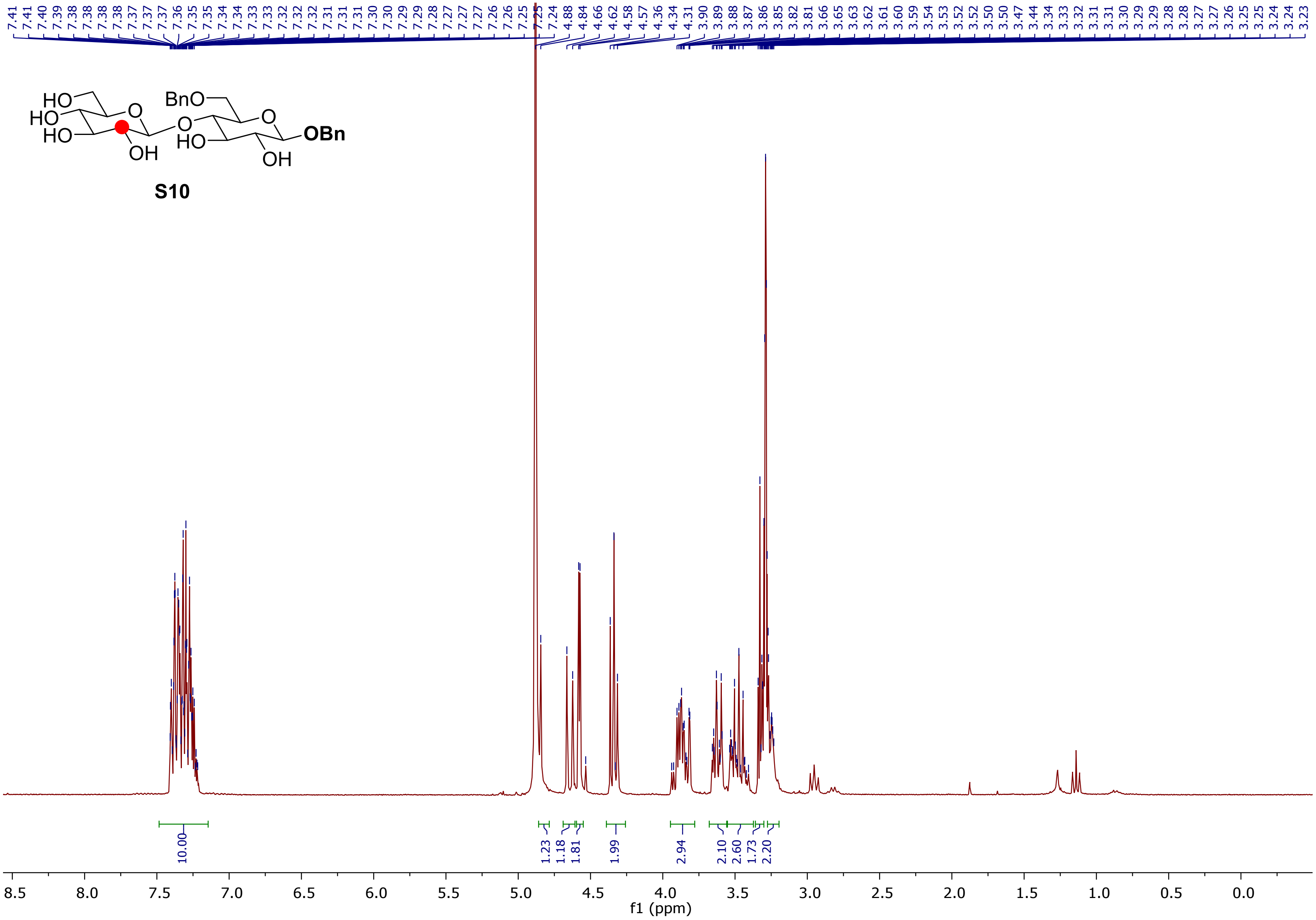
7.41 7.41 7.40 7.39 7.38 7.38 7.38 7.38 7.37 7.37 7.37 7.36 7.35 7.35 7.34 7.34 7.33 7.33 7.32 7.32 7.32 7.31 7.31 7.31 7.30 7.30 7.29 7.29 7.28 7.27 7.27 7.27 7.26 7.26 7.25 7.25 7.24 4.88 4.84 4.66 4.62 4.58 4.57 4.36 4.34 4.31 3.90 3.89 3.88 3.87 3.86 3.85 3.82 3.81 3.66 3.65 3.63 3.62 3.61 3.60 3.59 3.54 3.53 3.52 3.52 3.50 3.50 3.47 3.44 3.34 3.33 3.32 3.31 3.31 3.30 3.29 3.29 3.28 3.28 3.27 3.27 3.26 3.25 3.25 3.24 3.24 3.23
HO
HO
HO
O
OH
BnO
O
HO
O
OH
OBn
S10
10.00
1.23
1.18
1.81
1.99
2.94
2.10
2.60
1.73
2.20
8.5 8.0 7.5 7.0 6.5 6.0 5.5 5.0 4.5 4.0 3.5 3.0 2.5 2.0 1.5 1.0 0.5 0.0
f1 (ppm)

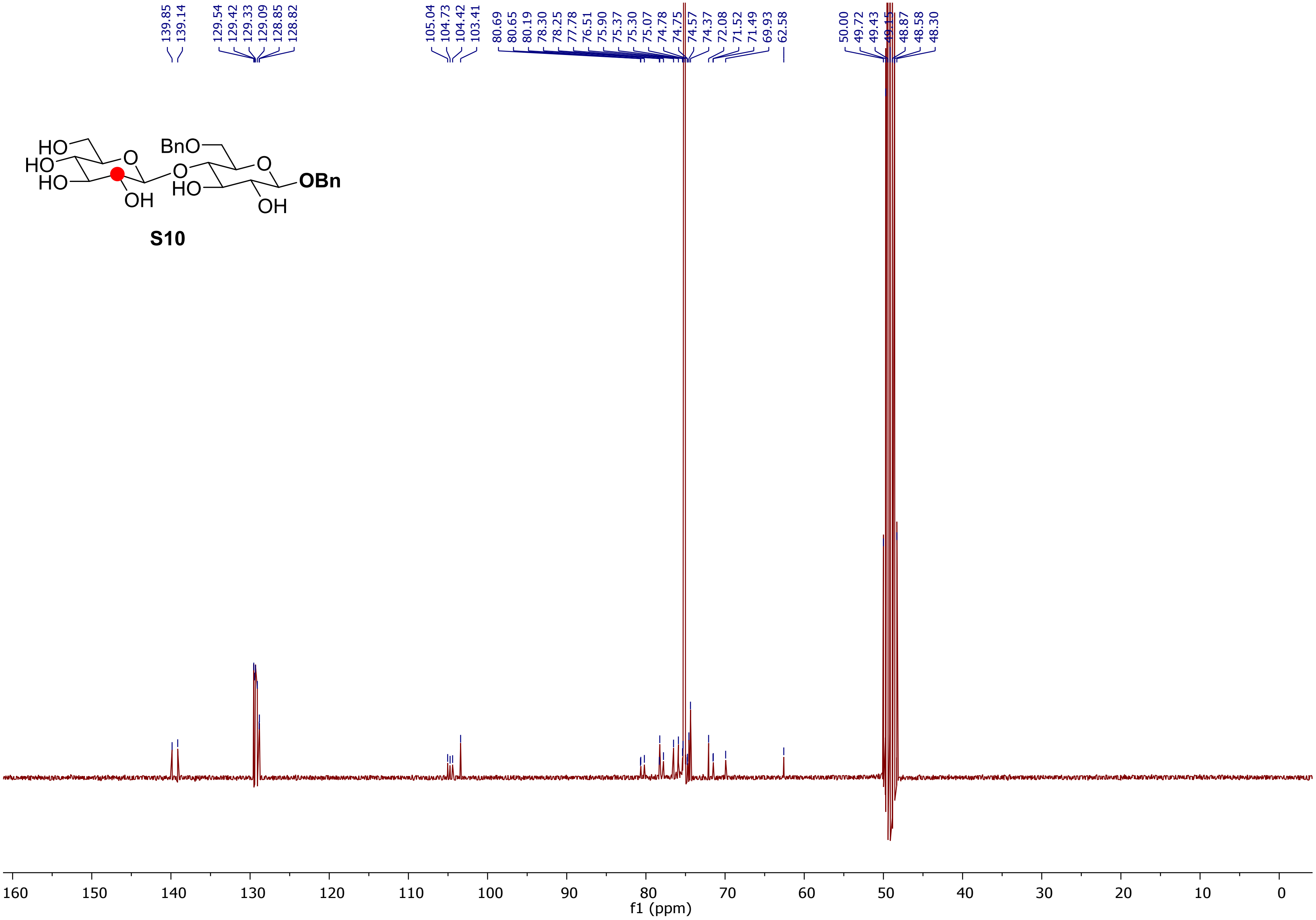
139.85
139.14
129.54
129.42
129.33
129.09
128.85
128.82
105.04
104.73
104.42
103.41
80.69
80.65
80.19
78.30
78.25
77.78
76.51
75.90
75.37
75.30
75.07
74.78
74.75
74.57
74.37
72.08
71.52
71.49
69.93
62.58
50.00
49.72
49.43
49.15
48.87
48.58
48.30
HO
HO
HO
OH
O
O
BnO
O
HO
OH
OBn
S10
160 150 140 130 120 110 100 90 80 70 60 50 40 30 20 10 0
f1 (ppm)

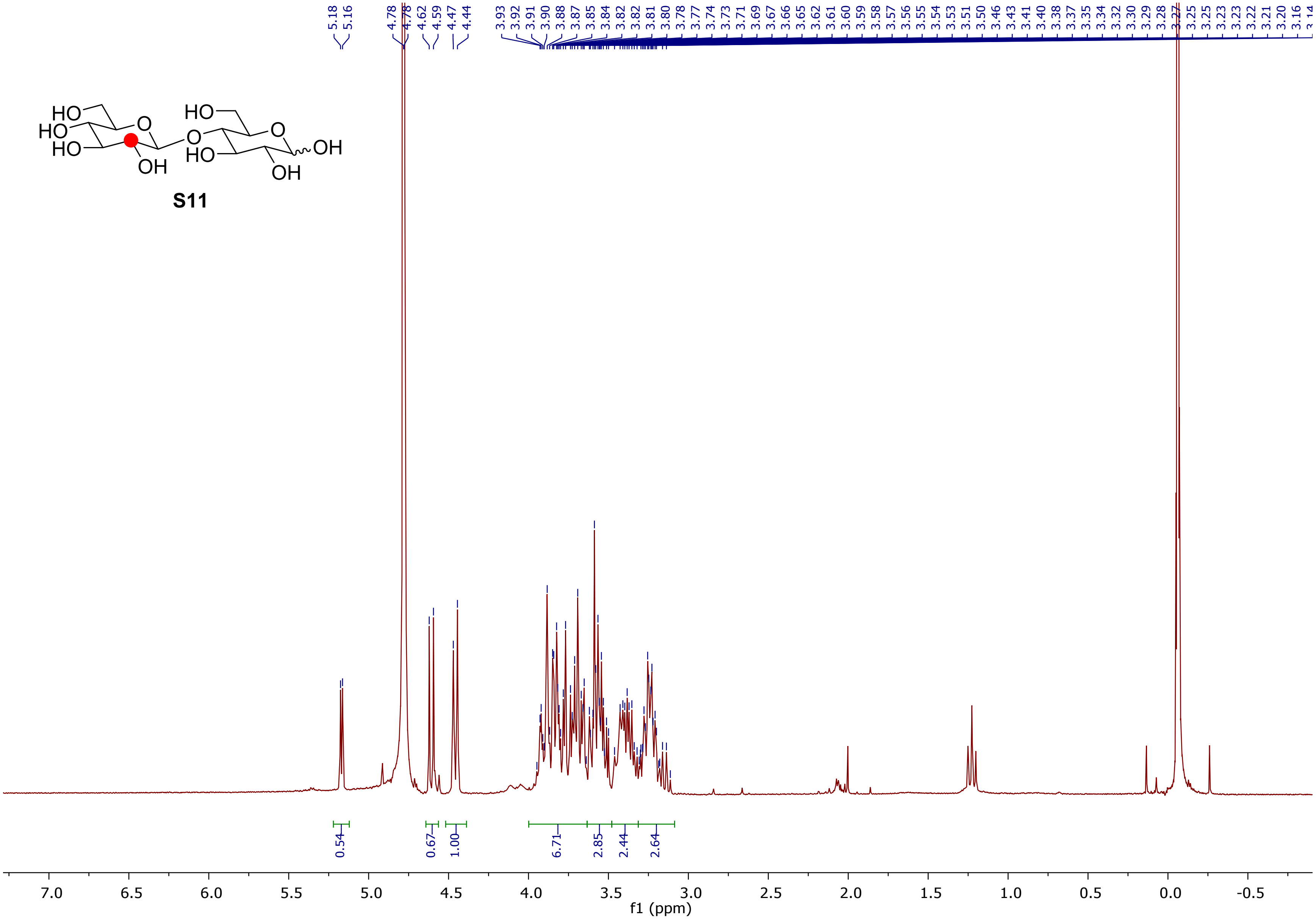

S11
f1 (ppm)

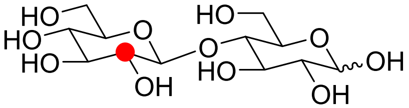
HO
HO
HO
O
OH
O
HO
HO
O
OH
OH

S11

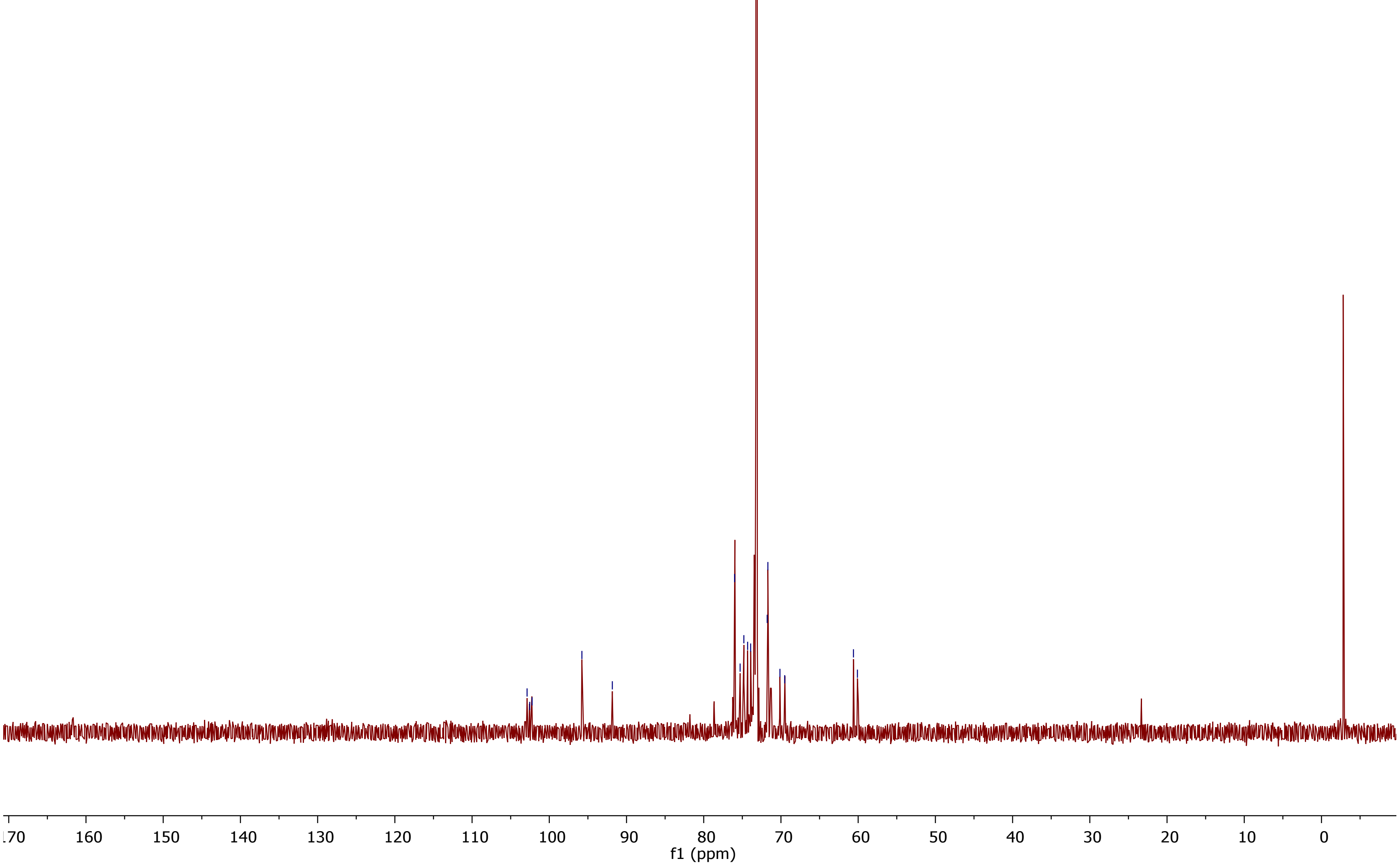
102.88
102.54
102.23
95.78
91.84
76.01
75.29
74.80
74.31
73.93
73.20
71.78
71.69
70.13
69.51
60.61
60.10
170
160
150
140
130
120
110
100
90
80
70
60
50
40
30
20
10
0
f1 (ppm)

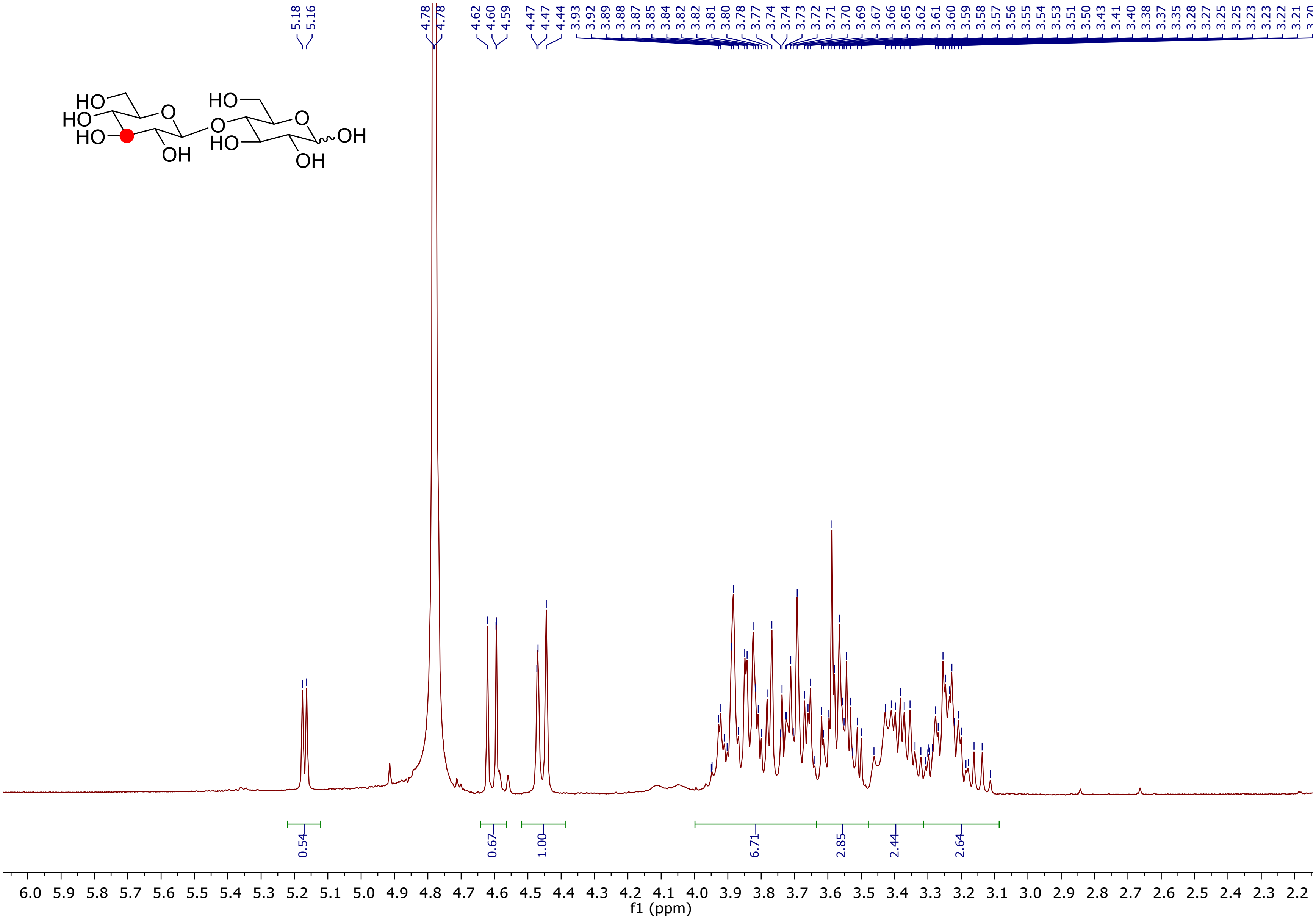
5.18
5.16
4.78
4.78
4.62
4.60
4.59
4.47
4.47
4.44
3.93
3.92
3.89
3.88
3.87
3.85
3.84
3.82
3.82
3.81
3.80
3.78
3.77
3.74
3.74
3.73
3.72
3.71
3.70
3.69
3.67
3.66
3.65
3.62
3.61
3.60
3.59
3.58
3.57
3.56
3.55
3.54
3.53
3.51
3.50
3.43
3.41
3.40
3.38
3.37
3.35
3.28
3.27
3.25
3.25
3.23
3.23
3.22
3.21
HO
HO
HO
O
OH
HO
O
O
HO
OH
OH
0.54
0.67
1.00
6.71
2.85
2.44
2.64
6.0 5.9 5.8 5.7 5.6 5.5 5.4 5.3 5.2 5.1 5.0 4.9 4.8 4.7 4.6 4.5 4.4 4.3 4.2 4.1 4.0 3.9 3.8 3.7 3.6 3.5 3.4 3.3 3.2 3.1 3.0 2.9 2.8 2.7 2.6 2.5 2.4 2.3 2.2
f1 (ppm)

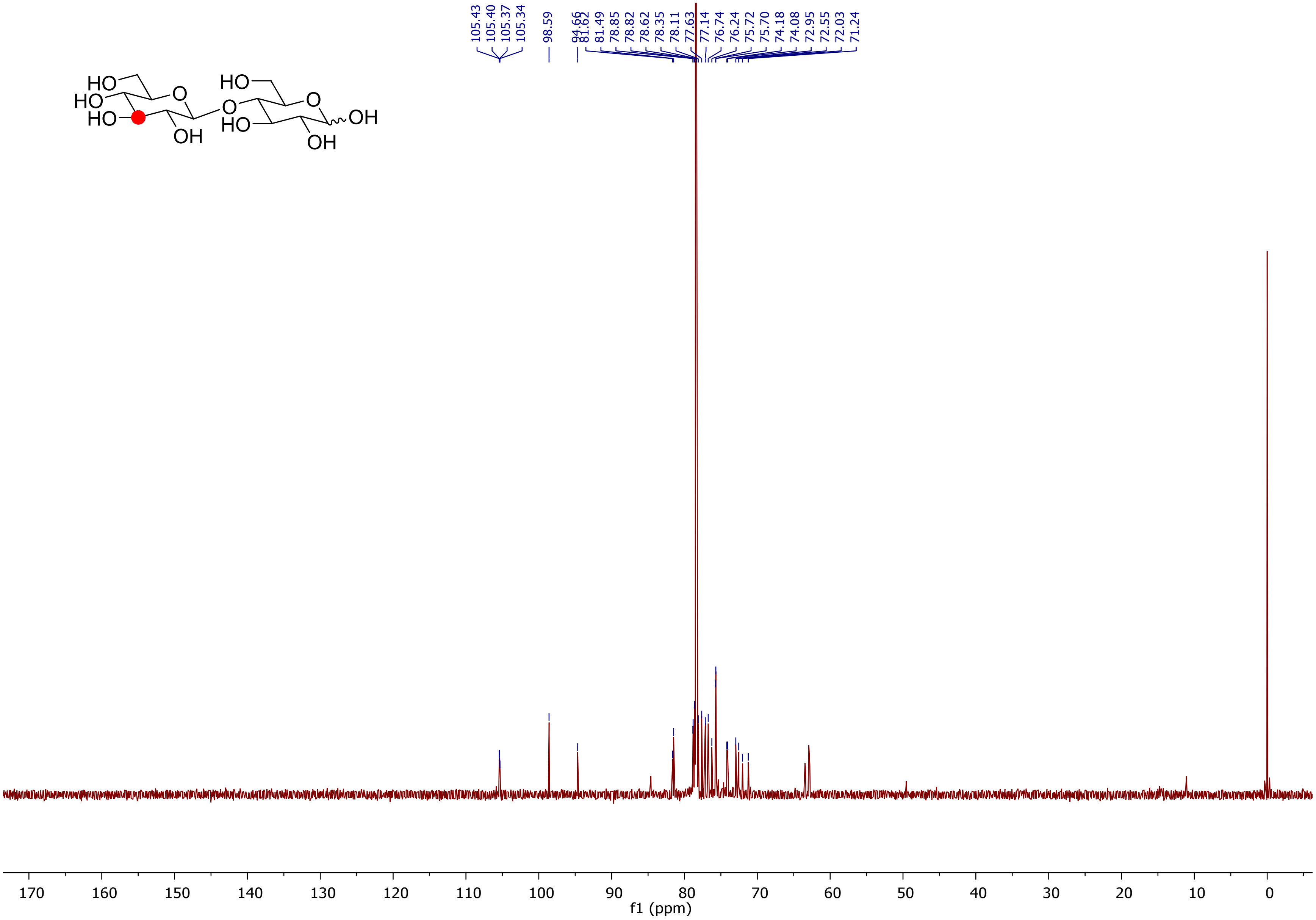

105.43
105.40
105.37
105.34
98.59
94.66
81.62
81.49
78.85
78.82
78.62
78.35
78.11
77.63
77.14
76.74
76.24
75.72
75.70
74.18
74.08
72.95
72.55
72.03
71.24
HO
HO
HO
O
OH
O
HO
O
HO
OH
OH
170
160
150
140
130
120
110
100
90
80
70
60
50
40
30
20
10
0
f1 (ppm)

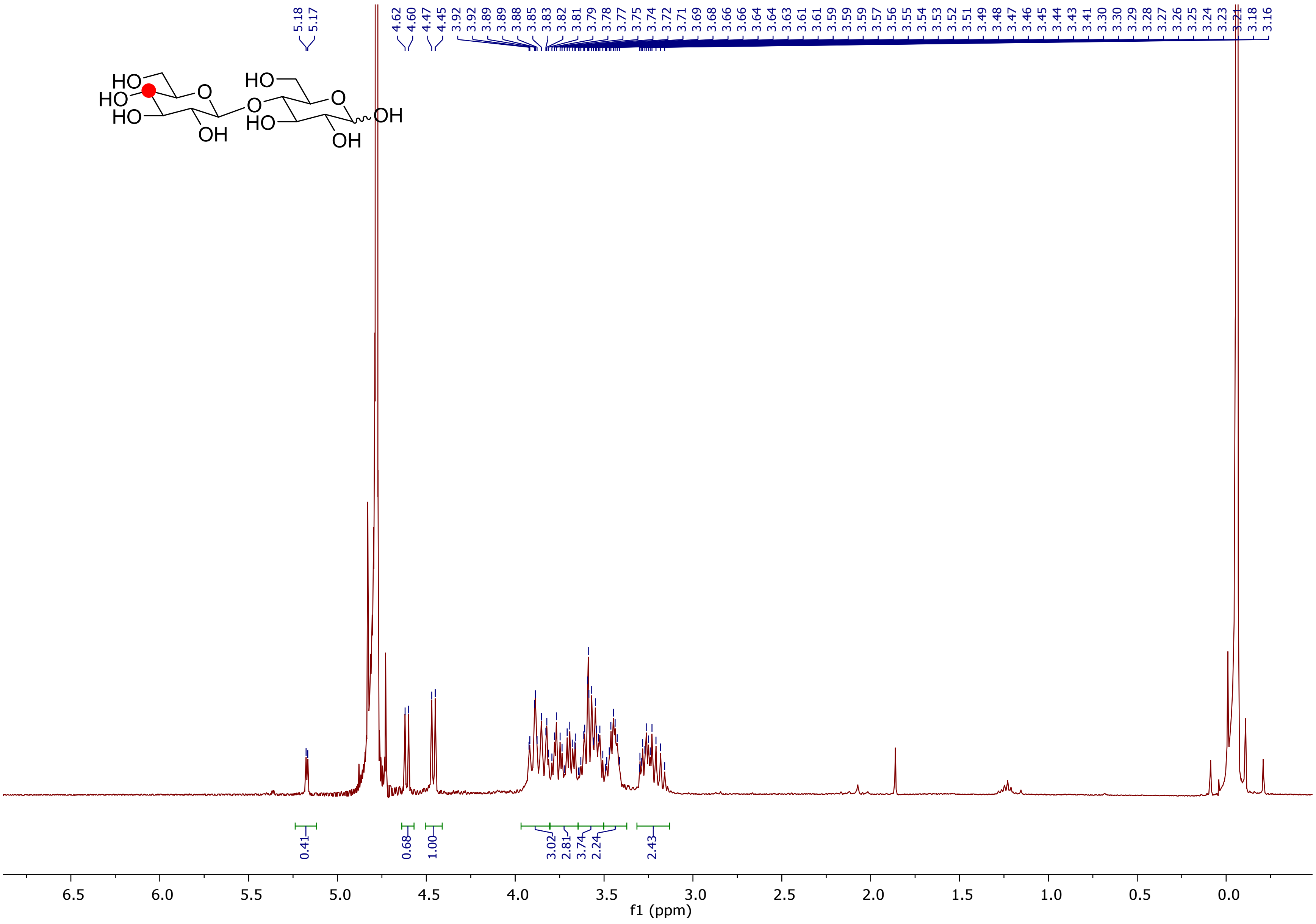

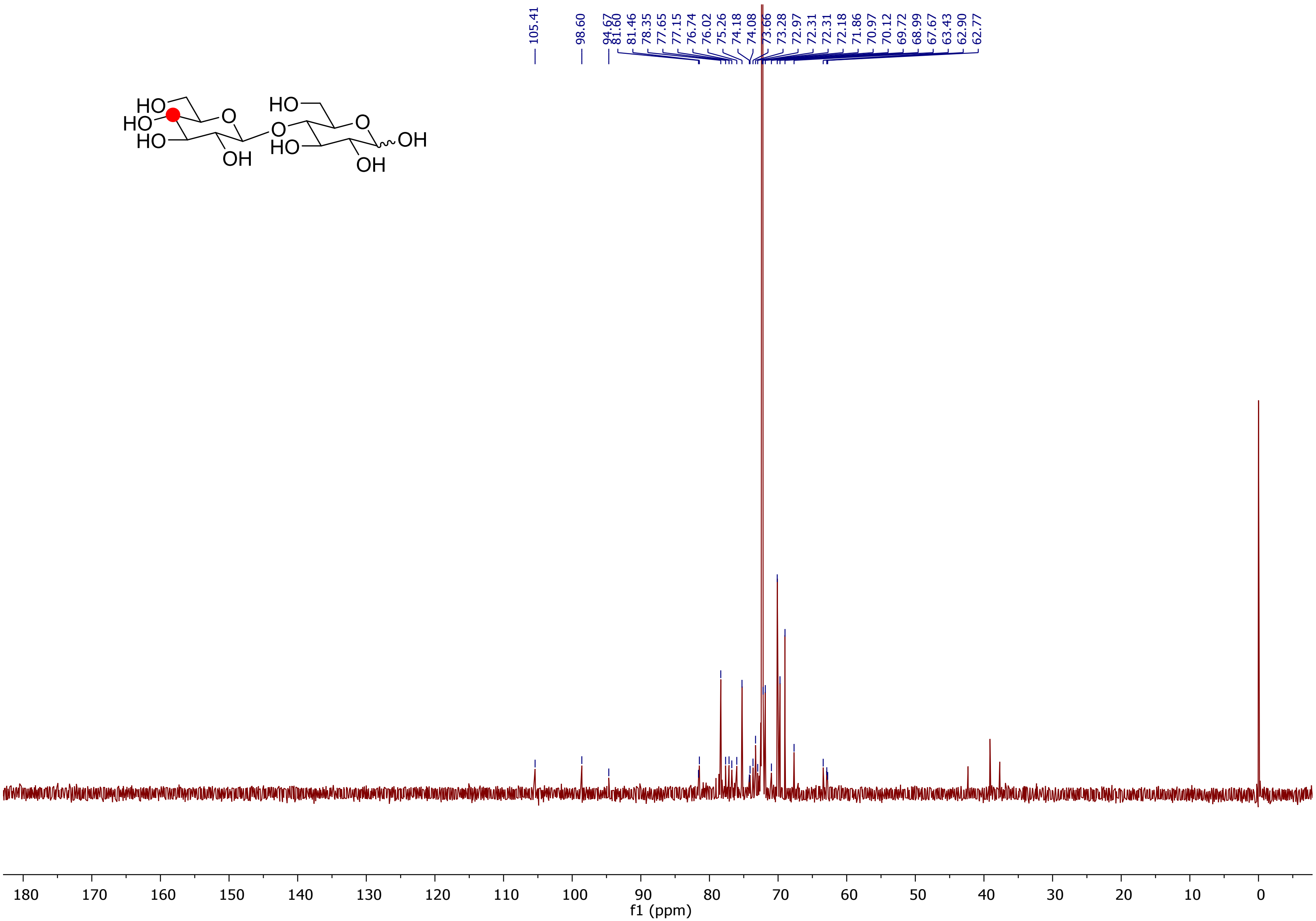
105.41
98.60
94.67
81.60
81.46
78.35
77.65
77.15
76.74
76.02
75.26
74.18
74.08
73.66
73.28
72.97
72.31
72.31
72.18
71.86
70.97
70.12
69.72
68.99
67.67
63.43
62.90
62.77
HO
HO
HO
O
OH
O
HO
HO
O
OH
OH
180
170
160
150
140
130
120
110
100
90
80
70
60
50
40
30
20
10
0
f1 (ppm)

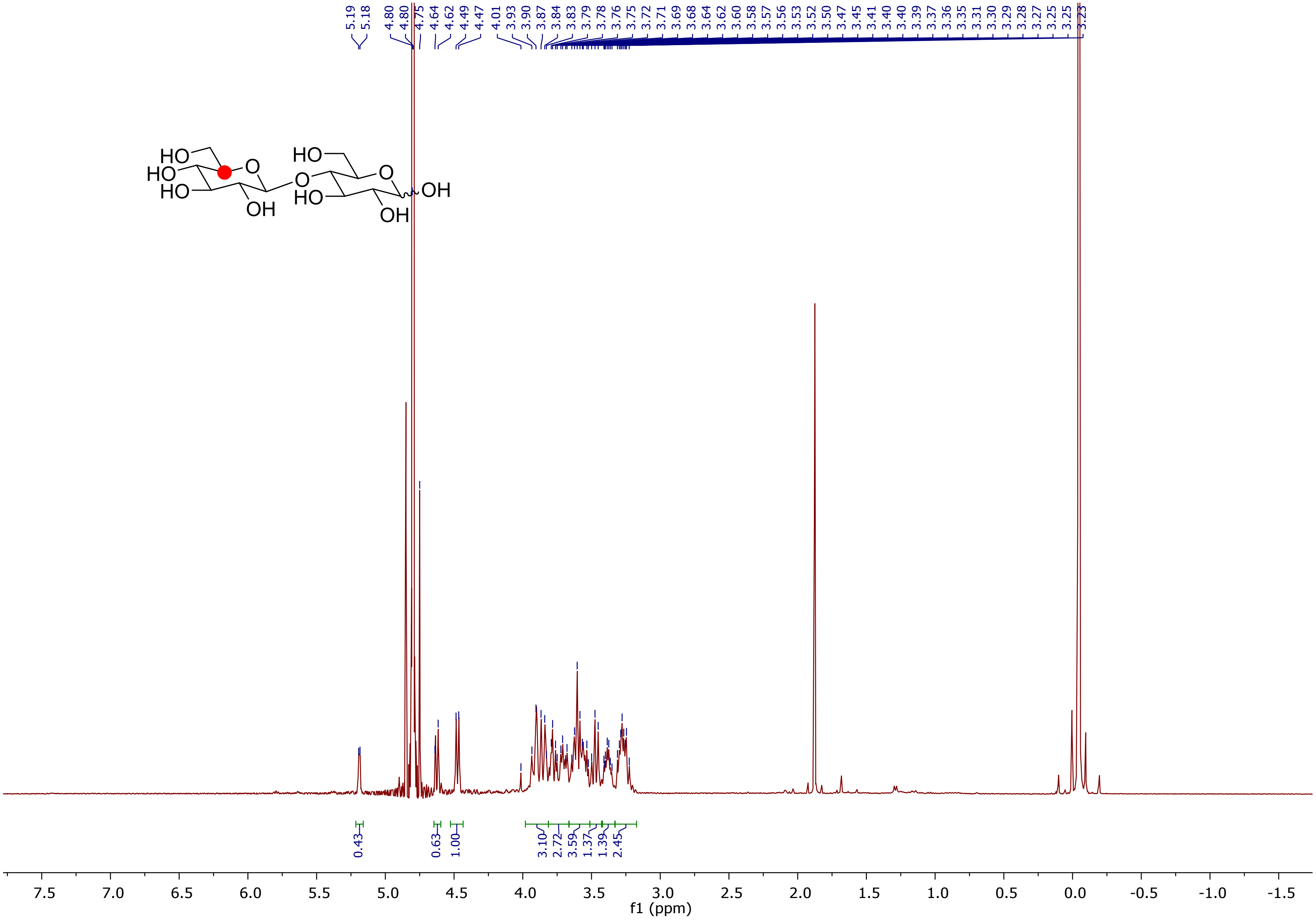
5.19
5.18
4.80
4.80
4.75
4.64
4.62
4.49
4.47
4.01
3.93
3.90
3.87
3.84
3.83
3.79
3.78
3.76
3.75
3.72
3.71
3.69
3.68
3.64
3.62
3.60
3.58
3.57
3.56
3.53
3.52
3.50
3.47
3.45
3.41
3.40
3.39
3.37
3.36
3.35
3.31
3.30
3.29
3.28
3.27
3.25
3.25
3.23
HO
HO
HO
O
OH
O
HO
O
HO
OH
OH
0.43
0.63
1.00
3.10
2.72
3.59
1.37
1.39
2.45
7.5
7.0
6.5
6.0
5.5
5.0
4.5
4.0
3.5
3.0
2.5
2.0
1.5
1.0
0.5
0.0
-0.5
-1.0
-1.5
f1 (ppm)

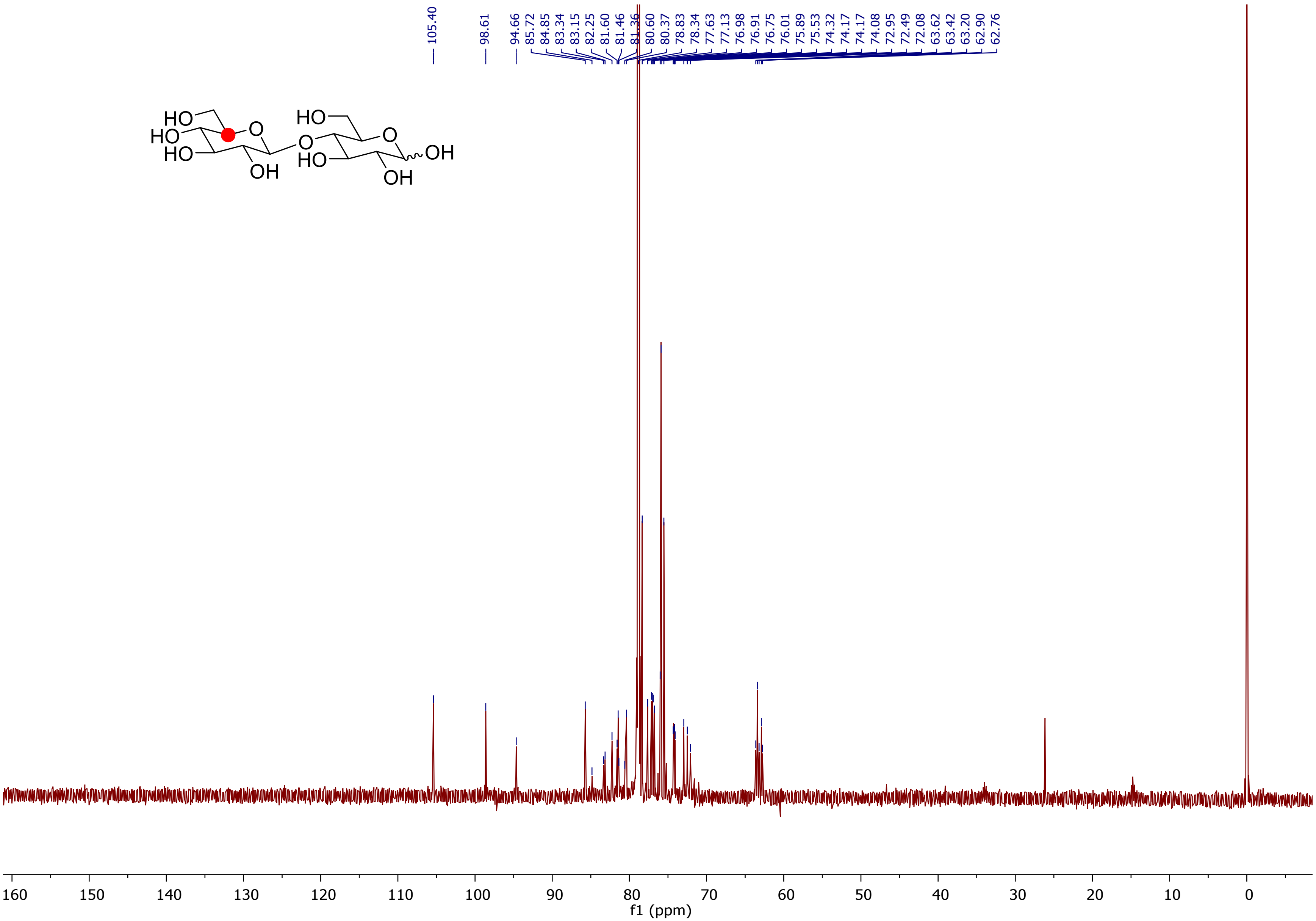
105.40
98.61
94.66
85.72
84.85
83.34
83.15
82.25
81.60
81.46
81.36
80.60
80.37
78.83
78.34
77.63
77.13
76.98
76.91
76.75
76.01
75.89
75.53
74.32
74.17
74.17
74.08
72.95
72.49
72.08
63.62
63.42
63.20
62.90
62.76
HO
HO
HO
O
OH
O
HO
O
HO
OH
OH
160
150
140
130
120
110
100
90
80
70
60
50
40
30
20
10
0
f1 (ppm)
